# Tunable Bifunctional Activity of Mn_*x*_Co_3−*x*_O_4_ Nanocrystals Decorated on Carbon Nanotubes for Oxygen Electrocatalysis

**DOI:** 10.1002/cssc.201800049

**Published:** 2018-03-26

**Authors:** Tingting Zhao, Srinivas Gadipelli, Guanjie He, Matthew J. Ward, David Do, Peng Zhang, Zhengxiao Guo

**Affiliations:** ^1^ Department of Chemistry University College London 20 Gordon Street London WC1H 0AJ UK; ^2^ CLS@APS Sector 20 Advanced Photon Source Canadian Light Source Inc., S44 Innovation Boulevard Saskatoon SK S7N 2V3 Canada; ^3^ Department of Chemistry Dalhousie University Halifax NS B3H 4R2 Canada

**Keywords:** doping, electrochemistry, nanostructures, spinel phases, transition metals

## Abstract

Noble‐metal‐free electrocatalysts are attractive for cathodic oxygen catalysis in alkaline membrane fuel cells, metal–air batteries, and electrolyzers. However, much of the structure–activity relationship is poorly understood. Herein, the comprehensive development of manganese cobalt oxide/nitrogen‐doped multiwalled carbon nanotube hybrids (Mn_*x*_Co_3−*x*_O_4_@NCNTs) is reported for highly reversible oxygen reduction and evolution reactions (ORR and OER, respectively). The hybrid structures are rationally designed by fine control of surface chemistry and synthesis conditions, including tuning of functional groups at surfaces, congruent growth of nanocrystals with controllable phases and particle sizes, and ensuring strong coupling across catalyst–support interfaces. Electrochemical tests reveal distinctly different oxygen catalytic activities among the hybrids, Mn_*x*_Co_3−*x*_O_4_@NCNTs. Nanocrystalline MnCo_2_O_4_@NCNTs (MCO@NCNTs) hybrids show superior ORR activity, with a favorable potential to reach 3 mA cm^−2^ and a high current density response, equivalent to that of the commercial Pt/C standard. Moreover, the hybrid structure exhibits tunable and durable catalytic activities for both ORR and OER, with a lowest overall potential of 0.93 V. It is clear that the long‐term electrochemical activities can be ensured by rational design of hybrid structures from the nanoscale.

## Introduction

Rechargeable metal–air batteries and regenerative fuel cells are highly desirable energy storage and conversion devices, in which the charge–discharge processes are critically defined by oxygen reduction and evolution reactions (ORR and OER, respectively). Often such reactions are catalyzed by platinum and iridium compounds.^1^, ^2^, ^3^ However, most metal–air batteries suffer from sluggish reaction kinetics, a relatively large overpotential, and unsatisfactory cyclic stability. Moreover, traditional ORR catalysts, such as platinum and its alloys, PtM (M=Pd, Au, Cu, Fe, Ni, Co, or W), are prone to poisoning by even a minute amount of impurity, for example, ppm levels of carbon monoxide in feed gas passivate the reaction in a few hours.^1^ Similarly, iridium and ruthenium oxides are the current benchmark catalysts for the OER.^4^, ^5^ Those monofunctional catalysts are also scarce. Therefore, it is of great significance to develop effective catalysts from earth‐abundant materials. Hence, transition‐metal/metal oxide nanoparticles and porous carbon nanostructures have received considerable attention, due to their high chemical and thermal stability, and flexibility for tuning of the surface and interface chemistry for the ORR, the OER, or bifunctionality. For example, attempts have been made in the synthesis of heteroatom‐doped carbon nanostructures (graphene, carbon nanotubes (CNTs), and carbon fibers),^6^, ^7^, ^8^ metal–organic framework (MOF)‐derived metal–N−C structures,^9^, ^10^, ^11^ perovskites,^12^, ^13^, ^14^, ^15^ and transition‐metal oxides (TMOs).^16^, ^17^, ^18^, ^19^, ^20^

TMOs, with their multiple oxidation states, show promising surface affinities for oxygen catalysis; the best examples are cobalt oxides. However, further efforts are needed to enhance their efficiency, reduce toxicity, and increase electronic conductivity. Part of the issues can be addressed by multicomponent metal oxides through improved electron hopping and enriched metal‐ion redox couples.^21^ Recently, more abundant and cost‐effective manganese‐based^22^ or combined cobalt–manganese complexes^19^, ^23^, ^24^, ^25^ have shown promise for electrocatalysis over other spinel oxides, such as NiCo_2_O_4_,^26^, ^27^, ^28^ CoFe_2_O_4_,^29^, ^30^, ^31^ and ZnCo_2_O_4_.^32^ Recent studies reveal that the spinel Mn_*x*_Co_3−*x*_O_4_ is a potential candidate, and the corresponding activities can be further tuned through its phase and composition.^2^, ^20^, ^25^, ^33^, ^34^, ^35^ The crystallographic phase structure of Mn_*x*_Co_3−*x*_O_4_ spinel is highly related to the Mn/Co ratio: a low Mn content (0≤*x*≤1.3) tends to form a cubic phase, whereas a high proportion of Mn favors a tetragonal structure.^25^ Such different phases lead to different electrocatalytic activities; the cubic and tetragonal spinel phases are beneficial for the ORR and OER, respectively, possibly due to subtle changes to the binding of relevant oxygen species at the surfaces.^2^, ^36^ The catalytic activity is closely associated with the elemental composition of manganese and cobalt, as well as the surface valence.^34^

Furthermore, different morphologies of TMOs, such as mesoporous nanoflakes,^37^ nanofibers,^20^ and porous microspheres,^38^, ^39^ have been considered to promote the overall kinetics of reactions with enhanced specific surface area for three‐phase contacts. These porous structures can provide interconnected channels for mass transport of both O_2_ and electrolyte necessary for sustaining the reactions. In addition, the conducting inorganic porous frameworks and carbon nanostructures are highly desirable substrate materials to further promote the catalysis reactions by enhancing the overall active sites and heterogeneous surface area.

An appropriate combination of TMOs and functionalized nanocarbons yield additional synergy for catalytic activity.^40^ There have been attempts to modify such carbon substrates by heteroatom doping, such as N‐doping to enhance the ORR,^41^, ^42^ and by induced defects as nucleation sites for anchoring TMO particles. For example, TMO nanoparticles anchored on nitrogen‐doped carbon structures show enhanced ORR and OER activities.^10^ Multiwalled CNTs with a well‐defined structure are highly conductive through the inner walls, whereas the outer walls can be functionalized for the anchoring of metal oxides. Ge et al. reported a dual‐phase Mn−Co−O/CNT‐based catalyst for both the ORR and OER that was synthesized through the direct combination of as‐prepared Mn−Co−O and CNTs in a hydrothermal process.^19^ Recently, Zhao et al. showed bifunctional activities of Mn−Co−O embedded in CNTs.^43^ However, the performance could be further improved by means of in situ synthesis to increase the density of active sites, for example, by reducing particle size, exposing active surface area, and strengthening the interaction between Mn−Co−O and CNTs. Further studies are also necessary to understand the specific roles played by the contributing structures and the optimum levels of the constituents, such as phase, crystallinity, and concentration of the TMO relative to that of the CNTs.

Herein, we show detailed structure–activity insights into Mn_*x*_Co_3−*x*_O_4_@NCNTs for both ORR and OER properties. To start with, the optimized structures were obtained by screening hybrids of nanocrystalline Mn_*x*_Co_3−*x*_O_4_@NCNTs that were synthesized directly on prefunctionalized CNTs with different combinations of constituents. The structures were further tailored by controlling the sizes and phase structures of the Mn_*x*_Co_3−*x*_O_4_ nanoparticles and surface modification of CNTs. Our synthesized Mn−Co−O was only 3–5 nm, which was much smaller than those synthesized by the ex situ method (≈50 nm), leading to much improved ORR and OER activities in terms of current density. The enhanced ORR limiting current density was almost twice that of Mn−Co−O embedded in CNTs. It also showed very high stability over long‐term operations for both the ORR and OER.

## Results and Discussion

**Scheme 1:**
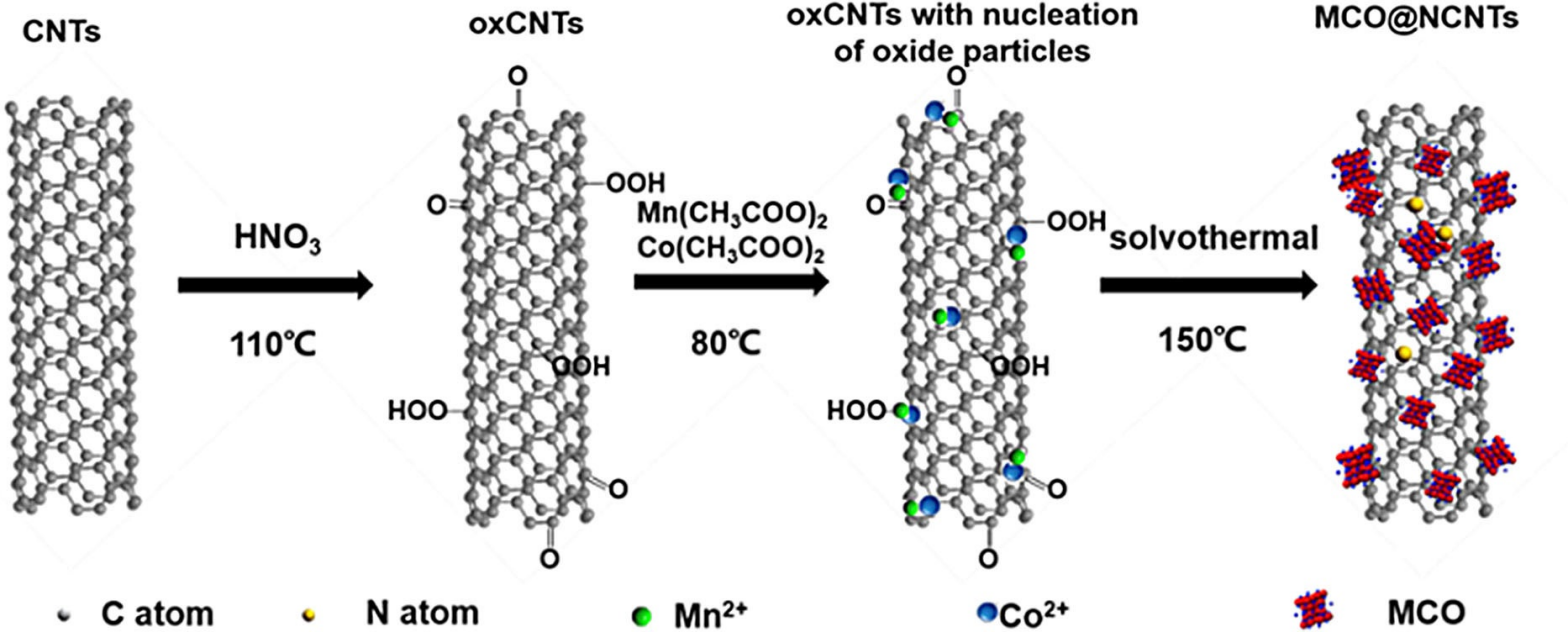
Synthetic process for a typical MCO@NCNTs sample, representing chemical functionalization of CNTs followed by surface anchoring of MCO nanocrystals.

**Figure 1:**
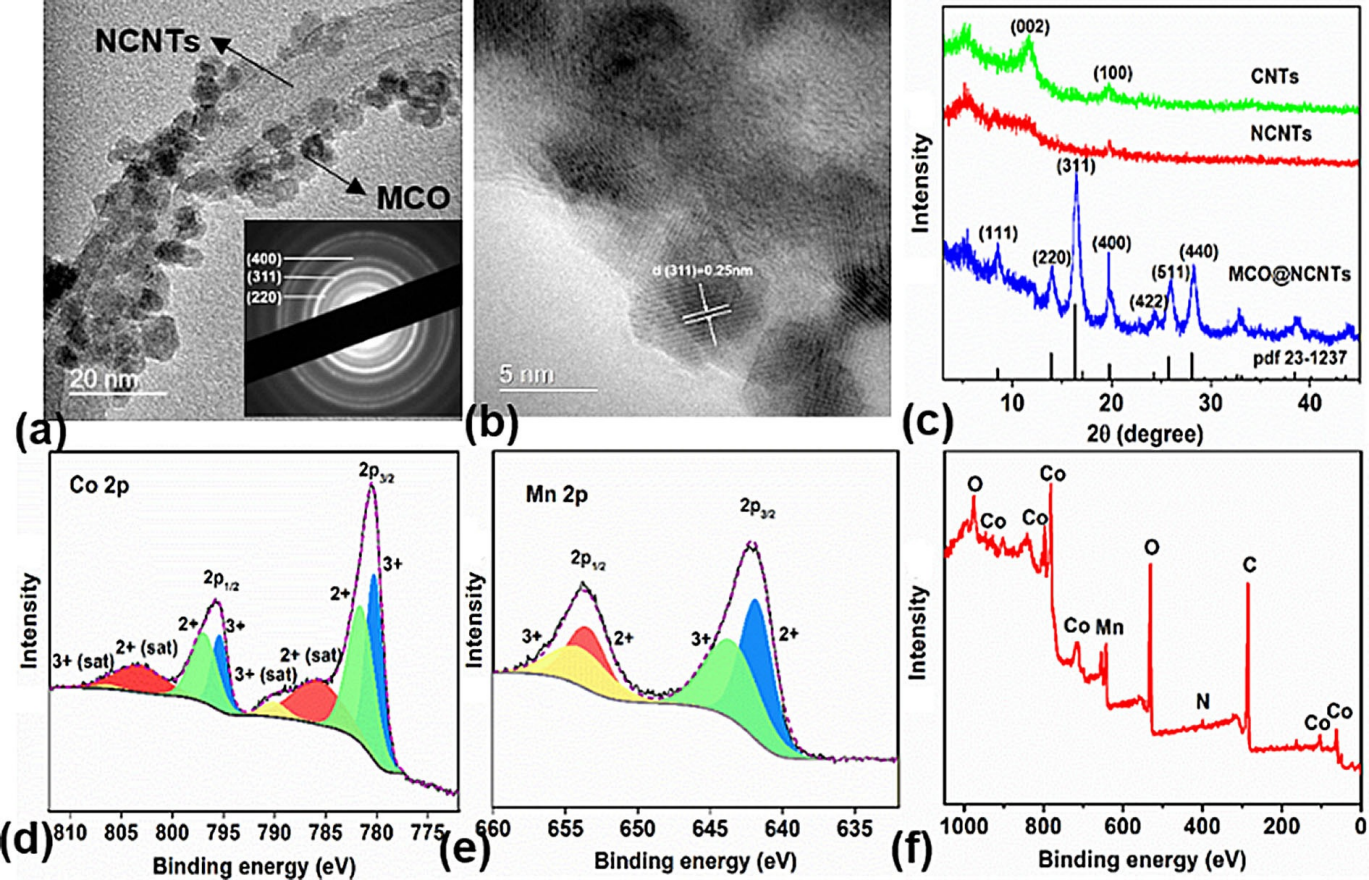
a) TEM image of MCO@NCNTs (inset: electron diffraction pattern). b) High‐resolution (HR) TEM image of MCO@NCNTs. c) Powder X‐ray diffraction (PXRD) results for CNTs, NCNTs, and MCO@NCNTs. X‐ray photoelectron spectroscopy (XPS) elemental analysis of MCO@NCNTs: core‐level spectra of Co 2p (d) and Mn 2p (e), and the survey spectrum (f).

A step‐by‐step synthetic route for MnCo_2_O_4_@NCNTs (MCO@NCNTs) hybrid structures is shown in Scheme 1. In a typical reaction, the as‐received CNTs were preoxidized (oxCNTs) to enrich functional surface oxy groups as anchoring sites for further functionalization and the growth of nanostructures. The hybrid MCO@NCNTs, in a desired composition and/or phase, were achieved by the nucleation and growth of MCO nanocrystals on the oxCNTs, from the hydrolyzed manganese and cobalt precursors, under a solvothermal process. Control over nucleation/growth of metal oxide species and nitrogen doping on the functional groups of the oxCNTs was simultaneously achieved by adjusting the pH with a solution of ammonia.^40^ Such synthesis yielded an extensive formation of MCO nanoparticles (of 3–5 nm) over the CNTs without aggregation, as shown in the TEM image in Figure 1 a. Electron diffraction (Figure 1 a, inset) shows that the MCO nanoparticles are in good agreement with the designed composition. The lattice fringes of about 0.25 nm for the nanoparticles observed under HRTEM in Figure 1 b are consistent with the *d* spacing of the (311) lattice plane of MnCo_2_O_4_. PXRD patterns (Figure 1 c) demonstrate the single‐phase nature of cubic spinel MnCo_2_O_4_ (JCPDS no. 23‐1237). Energy‐dispersive X‐ray spectroscopy (EDS) elemental mapping further shows evenly distributed Mn and Co, with an atomic ratio of about 1:2 in the MCO@NCNTs (Figure S1 in the Supporting Information). Furthermore, characteristic +2 and +3 oxidation states of Co and Mn in MCO are identified by XPS (Figure 1 d and e).^20^ The survey spectrum yields a Co/Mn atomic ratio of about two, which is consistent with the EDS results (Figure S1 in the Supporting Information). The surface oxidation of the CNTs can be illustrated by the characteristic C 1s peaks for C−O and O−C=O groups. Nitrogen doping of CNTs accounts for about 1.4 at %, predominantly in pyridinic and pyrrolic N sites (Figure S2 in the Supporting Information).

**Figure 2:**
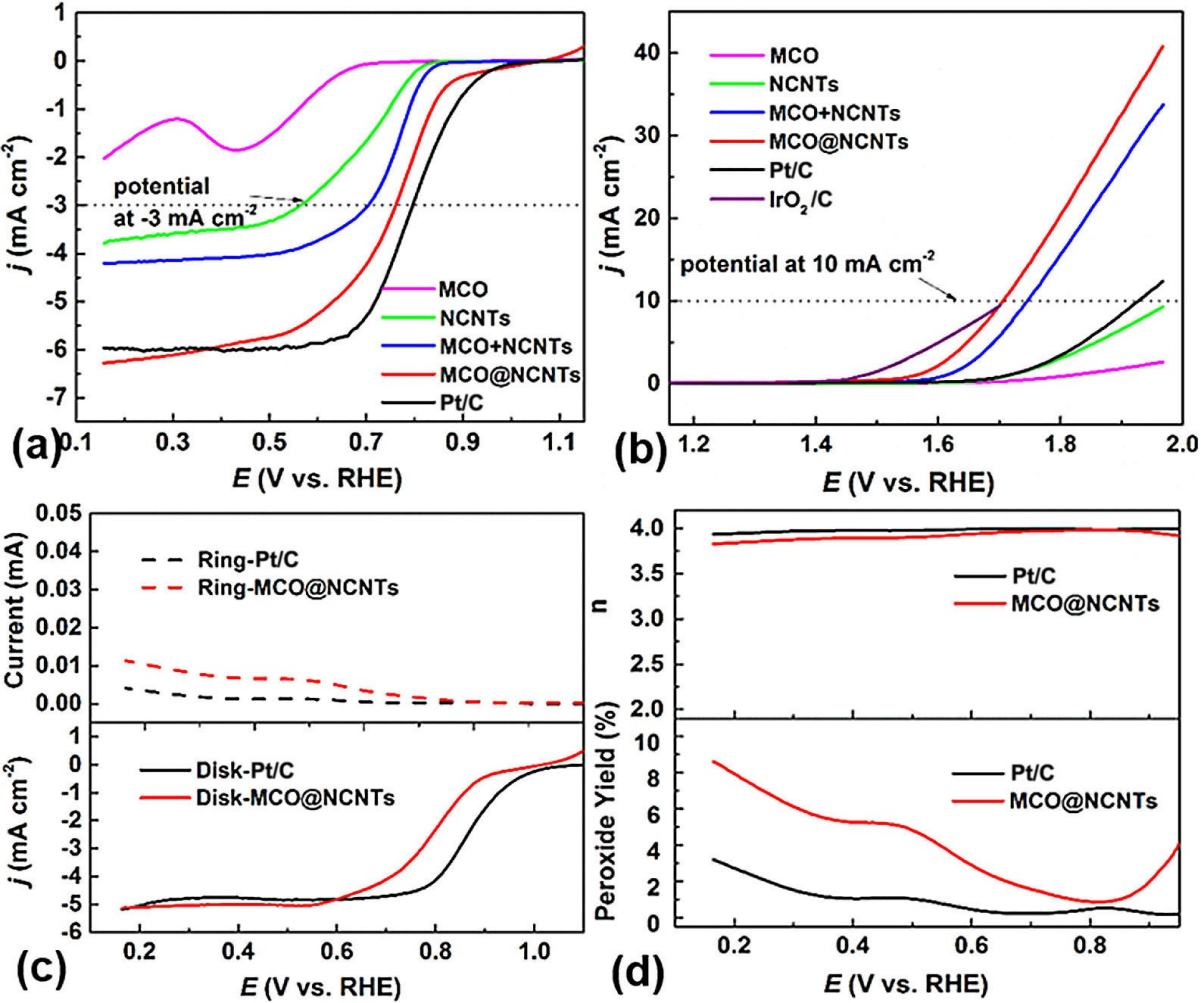
a) ORR and b) OER LSV curves of MCO, NCNTs, MCO+NCNTs, MCO@NCNTs, and reference standards Pt/C and IrO_2_/C,^49^ on a rotating disk electrode (RDE) measured in O_2_‐saturated 0.1 m KOH at a scan rate of 10 mV s^−1^ and 1600 rpm (at room temperature, without *IR* compensation). c) Comparative ORR LSV curves of MCO@NCNTs and Pt/C on a rotating ring disk electrode (RRDE) in O_2_‐saturated 0.1 m KOH at 1600 rpm. The disk potential was scanned at 10 mV s^−1^ by maintaining the ring at 1.46 V versus a reversible hydrogen electrode (RHE). d) Percentage of peroxide formation (bottom) with respect to the total oxygen reduction products and electron‐transfer number, *n* (top), of MCO@NCNTs and Pt/C at various potentials based on the corresponding RRDE data.

Electrocatalytic activity of the hybrid sample, MCO@NCNTs, for the ORR is evaluated by cyclic voltammetry (CV) and linear sweep voltammetry (LSV; Figures S3 and S4 in the Supporting Information and Figure 2). For a comparison of performance, the commercial standard, Pt/C; the individual counterparts of MCO and NCNTs; and their physical mixture of equivalent mass composition to the MCO@NCNTs were also tested under identical conditions to those used for the hybrid sample. A more positive potential to achieve a reference current density, commonly at 3 mA cm^−2^, is more desirable for an effective ORR catalyst. As noted from Figure 2 a, the MCO@NCNTs show a comparable ORR activity to that of commercial Pt/C. The Tafel slopes further prove their kinetic similarity (Figure S5 in the Supporting Information). Moreover, a gradual performance improvement is also noted from Figure 2 a in the following order: MCO<NCNTs<MCO+NCNTs (the physical mixture of the two)<MCO@NCNTs (the hybrid). It is also noted in Figure 2 a that the observed limiting current densities of the samples vary considerably, although in a similar order as that for their potentials. In theory, the limiting current density at a very high overpotential is dominated by mass transfer and should be at similar levels for a given type of catalyst that involves the same rate‐controlling step and electron‐transfer mechanism, as often described by the Koutecky–Levich equation. However, for different types of catalysts, as in the current case, the apparent limiting current can vary due to different properties of the catalysts, that is, the rate‐controlling step and/or the specific electron‐transfer mechanism(s), in relation to the specific active site and active site density (or loading level), before the diffusion‐limiting term sets in. Such a phenomenon is also reported in the literature.^1^, ^2^, ^3^

In the case of the OER, a less positive potential to achieve a reference current density, for example, of 10 mA cm^−2^, is more desirable for the catalyst.^44^, ^45^ Clearly, the MCO@NCNTs hybrids exhibit a relatively high OER activity (Figure 2 b), which is close to that of the benchmark material, IrO_2_/C (Figure S6 in the Supporting Information).^46^ It is worth noting that the OER activity of Pt/C is rather low, due to passivation by oxide formation, which hinders electron transfer.^47^, ^48^ Also, both the NCNTs and MCO are poor OER performers that require a relatively large overpotential. The physical mixture of MCO+NCNTs is also inferior to that of the MCO@NCNTs hybrids.

RRDE measurements further reveal that the ORR mechanism involves a direct four‐electron or intermediate (peroxides)‐mediated two‐electron process. Ring and disk current responses directly indicate the peroxide yield and electron‐transfer reaction steps (Figure 2 c and d). Notably, the MCO@NCNTs sample exhibits a comparable disk current to that of Pt/C, which is in good agreement with the RDE results. A slightly higher ring current indicates more peroxide oxidation, about 9 % compared with about 5 % in Pt/C. Therefore, the hybrid sample also shows a fast, four‐electron pathway, with an average electron‐transfer number equal to 3.9.

Bifunctionality of the catalysts is characterized by the difference in potentials between the two metrics for the ORR and OER (Δ*E*=*E*_OER_−*E*_ORR_), at which the respective current densities of −3 and 10 mA cm^−2^ are attained.^46^ A smaller potential difference indicates more efficient bifunctional performance of the catalyst. As summarized in Table 1, MCO@NCNTs exhibit the lowest potential difference of 0.94 V.

**Table 1:** A comparison of the bifunctional activities of MCO@NCNTs and their counterparts.

Sample	*E*_reaction_ [V]		Δ*E*
	ORR benchmark^[a]^	OER benchmark^[b]^	[V]
MCO	<0.1	>1.9	>2
NCNTs	0.56	≈1.00	≈1.40
MCO+NCNTs	0.70	1.74	1.04
MCO@NCNTs	0.76	1.70	0.94
Pt/C	0.79	1.92	1.13

[a] At *j*=−3 mA cm^−2^. [b] At *j*=10 mA cm^−2^.

The catalytic properties of hybrid nanostructures are further investigated with respect to the compositions, phases, and sizes of the Mn_*x*_Co_3−*x*_O_4_ nanocrystals at CNTs. Several hybrids, Mn_*x*_Co_3−*x*_O_4_@NCNTs, with *x=*0, 1, 1.5, 2, and 3, were prepared under identical synthetic conditions to yield hybrids of NCNTs with Co_3_O_4_, MnCo_2_O_4_, Mn_1.5_Co_1.5_O_4_, Mn_2_CoO_4_, and Mn_3_O_4_, respectively. PXRD results show that all samples are well crystallized with either a cubic (for *x*=0 and 1) or tetragonal (for *x*≥1.5) phase (Figure S7 in the Supporting Information). Diffraction patterns with characteristic peak widths and intensities further suggest high crystallinity/large crystal growth, particularly if the manganese substitution for cobalt is ≥2. The crystallinity and phase structure of the samples are confirmed by TEM and electron diffraction (Figures S4 b–e and S5 in the Supporting Information). Notably, increasing the Mn/Co ratio leads to a considerable increase in particle size from 3–5 nm for Co_3_O_4_ or MnCo_2_O_4_, to 10–20 nm for Mn_1.5_Co_1.5_O_4_ and Mn_2_CoO_4_, and up to about 50 nm for Mn_3_O_4_.

**Figure 3:**
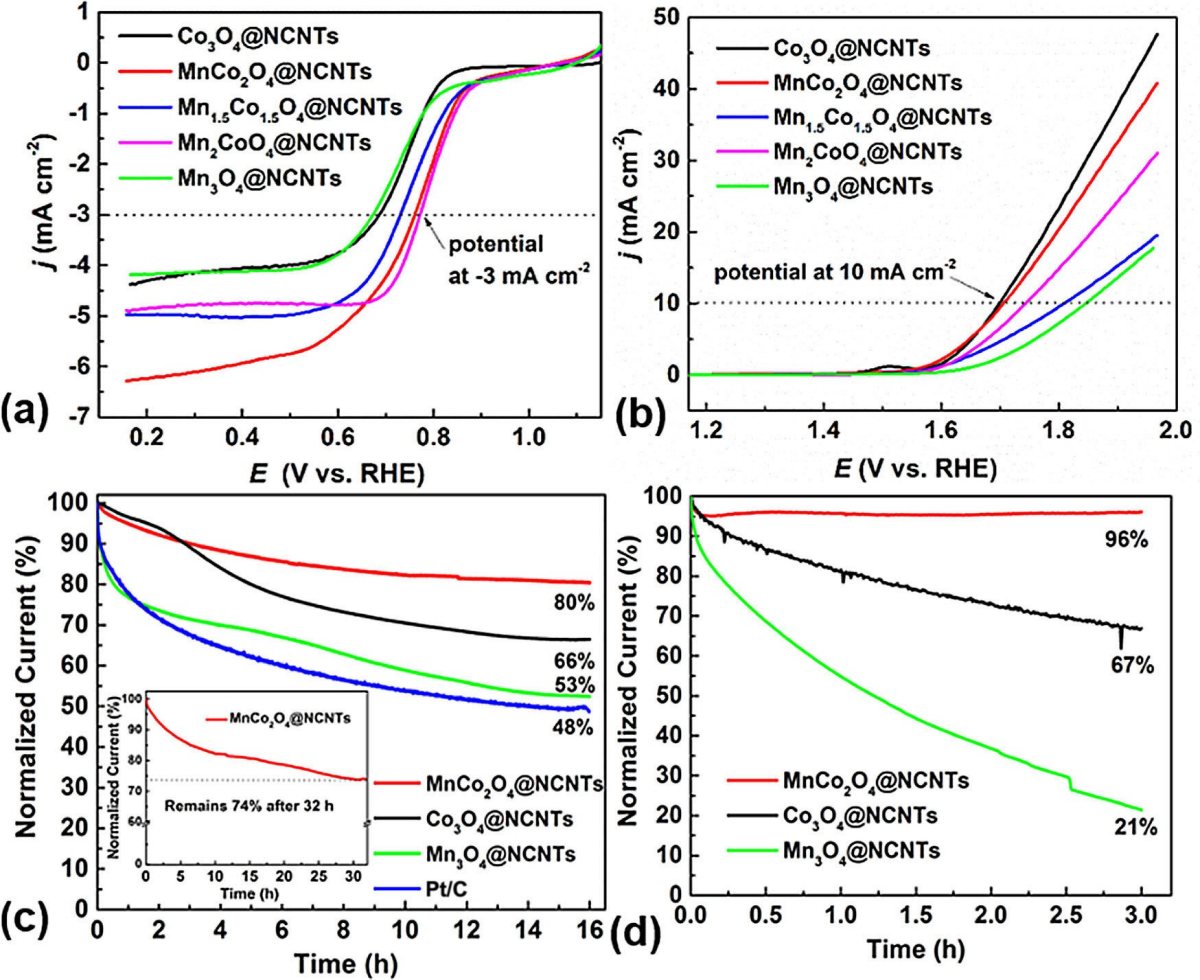
Electrocatalytic activity performance of Mn_*x*_Co_3−*x*_O_4_@NCNTs on a glassy carbon electrode measured in O_2_‐saturated 0.1 m KOH. LSV curves of the a) ORR and b) OER at a scan rate of 10 mV s^−1^ and 1600 rpm. Chronoamperometry (CA) response curves of c) the ORR at 0.66 V versus RHE under 1600 rpm (inset: MnCo_2_O_4_@NCNTs for prolonged operation of 32 h) and d) the OER at 1.66 V versus RHE under 2500 rpm.

Such diversity in crystal sizes, phase structures, and compositions leads to a considerable change in catalytic performance (Figure 3 a and Figures S7–S10 in the Supporting Information). Indeed, a highly enhanced ORR activity is observed in composites, Mn_*x*_Co_3−*x*_O_4_@NCNTs (1≤*x*≤2), compared with those of Co_3_O_4_@NCNTs and Mn_3_O_4_@NCNTs. For instance, despite exhibiting similar crystallite sizes (3–4 nm) and cubic‐phase structures, the MnCo_2_O_4_ sample shows a highly favorable reduction potential to reach 3 mA cm^−2^ and a large current response over that of Co_3_O_4_. The ORR catalytic activity is reduced at higher manganese concentrations; this is directly attributed to the effect of both larger size and less favorable activity of the tetragonal phase of the nanoparticles. A similar activity trend is observed for the OER (Figure 3 b), for which ultrasmall crystals of cubic‐phase Co_3_O_4_ or MnCo_2_O_4_ structures at NCNTs exhibit the highest activity.

In addition to the lowest possible potential to achieve a high current response, the viability of such catalysts also depends on their stability during continuous operation. An ideal catalyst should exhibit a constant current response without significant loss under a constant applied potential, or vice versa. However, this is one of the major drawbacks for the commercial Pt/C standard (Figure 3 c). A considerable drop in activity, up to 50 %, is observed after 15 h of continuous operation.^50^ In contrast, the MnCo_2_O_4_@NCNTs are highly stable, maintaining about 80 % of the initial current under similar experimental conditions, and still showing about 74 % current retention after 32 h of continuous operation. Relatively large activity losses of up to 34 and 47 % are observed for the Co_3_O_4_@NCNTs and Mn_3_O_4_@NCNTs, respectively. A similar activity/durability trend is also observed for the OER (Figure 3 d). There is negligible performance loss for the MnCo_2_O_4_@NCNTs, whereas 30 to 80 % drop in the current response is seen for Co_3_O_4_@NCNTs and Mn_3_O_4_@NCNTs. The comparatively low stability of Mn_3_O_4_@NCNTs for the OER may be attributed to the relatively large particles, which are prone to detachment from the surface of CNTs, due to geometrical and thermomechanical mismatches, during the evolution of O_2_ from the hydroxyl groups, leading to a drop in stability for the OER. Clearly, the partial substitution of manganese for cobalt stabilizes the bifunctional activities for both the ORR and OER, which may be directly attributed to the increased valence states of Mn and Co, and the formation of new metal‐ion redox couples (see CV curves, Figure S6 in the Supporting Information, and also below).

**Figure 4:**
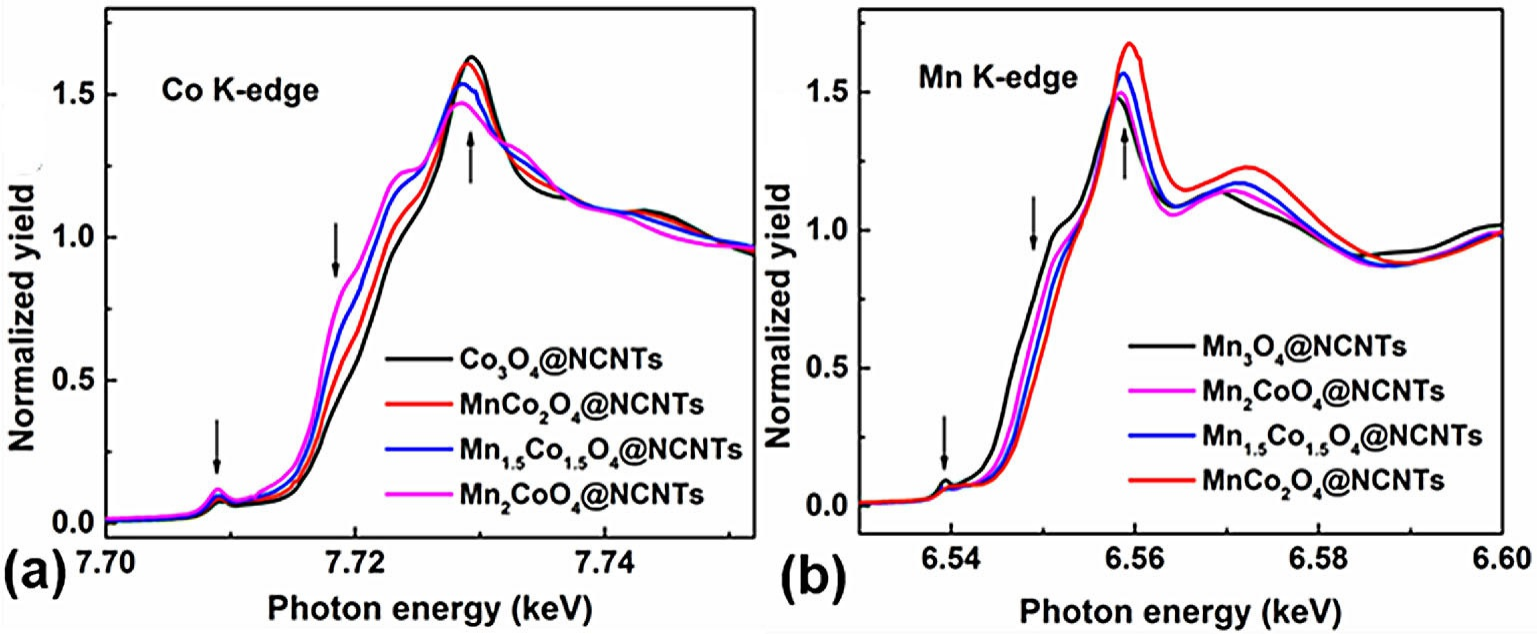
a) Co and b) Mn K‐edge of Mn_*x*_Co_3−*x*_O_4_@NCNTs, with *x=*0, 1, 1.5, 2, and 3, from XANES.

To understand this activity and stability trend of the Mn_*x*_Co_3−*x*_O_4_@NCNTs, the structures were further probed by Co‐ and Mn‐edge X‐ray adsorption near‐edge structure (XANES) to identify the valence change with the variation of Mn/Co ratio (Figure 4). XANES results show clear changes in both Co and Mn K edges with respect to concentration, which implies that the dopants consistently modify the structure and bonding of the metal oxide nanoparticles (see arrows in Figure 4). It can be seen that the Co K‐edge XANES of Mn_*x*_Co_3−*x*_O_4_ samples exhibit a similar spectral shape to that of the Co_3_O_4_ reference; this indicates that these three samples contain mixed‐valence states of Co^3+^ and Co^2+^ species, similar to the Co_3_O_4_ reference. It is known that the most intense absorption feature at about 7730 eV is sensitive to the oxidation state of Co, and its positive shift suggests the enhanced ratio of Co^3+^/Co^2+^ with decreasing Mn/Co ratio.^51^ A similar trend is also seen in the Mn K edge (Figure 4 b), for which there is an absorption feature at about 6560 eV; this again suggests increased population of Mn^3+^/Mn^2+^ with decreasing Mn/Co ratio in the samples. This may be explained as follows. Due to the difference in electronegativity (electron affinity), charge redistribution occurs after Mn doping in Co_3_O_4_—the less electronegative species (here Mn) will “donate” electron density towards the more electronegative species (here Co), while the overall charge neutrality is maintained in Mn_*x*_Co_3−*x*_O_4_ without any significant change to the oxygen content, that is, the total positive charge can be maintained at 8+, to counterbalance the 4×2− charge from O. In other words, the doping of Mn in Co_3_O_4_ may cause the (average) valence of Co to decrease, relative to that in pure Co_3_O_4_, whereas it will cause the valence of doped Mn to increase, compared with that in pure Mn_3_O_4_. Hence, partial substitution of Co^3+^/Co^2+^ by Mn^3+^/Mn^2+^ can enrich the metal‐ion redox pairs in the hybrids, thus leading to enhanced activities and long‐term stability for both the ORR and OER. Moreover, manganese doping may also lead to the creation of oxygen vacancies close to the doping sites, which, in turn, promote molecular sorption on catalyst surfaces to facilitate ORR/OER activities. A recent study showed that the intrinsic ORR/OER activity of MnCo_2_O_4_ was a function of the Mn valence on the octahedral sites through its influence on the antibonding orbital occupancy of the Mn−O bond.^52^ Experimental and theoretical (through the manipulation of the binding strength of ORR/OER intermediates governed by e_g_ filling) results suggest that MnCo_2_O_4_ and Mn_3_O_4_ are the best candidates for the ORR and OER, respectively. These studies are consistent with our optimum ORR activity performance observed in the MnCo_2_O_4_@NCNTs.

**Figure 5:**
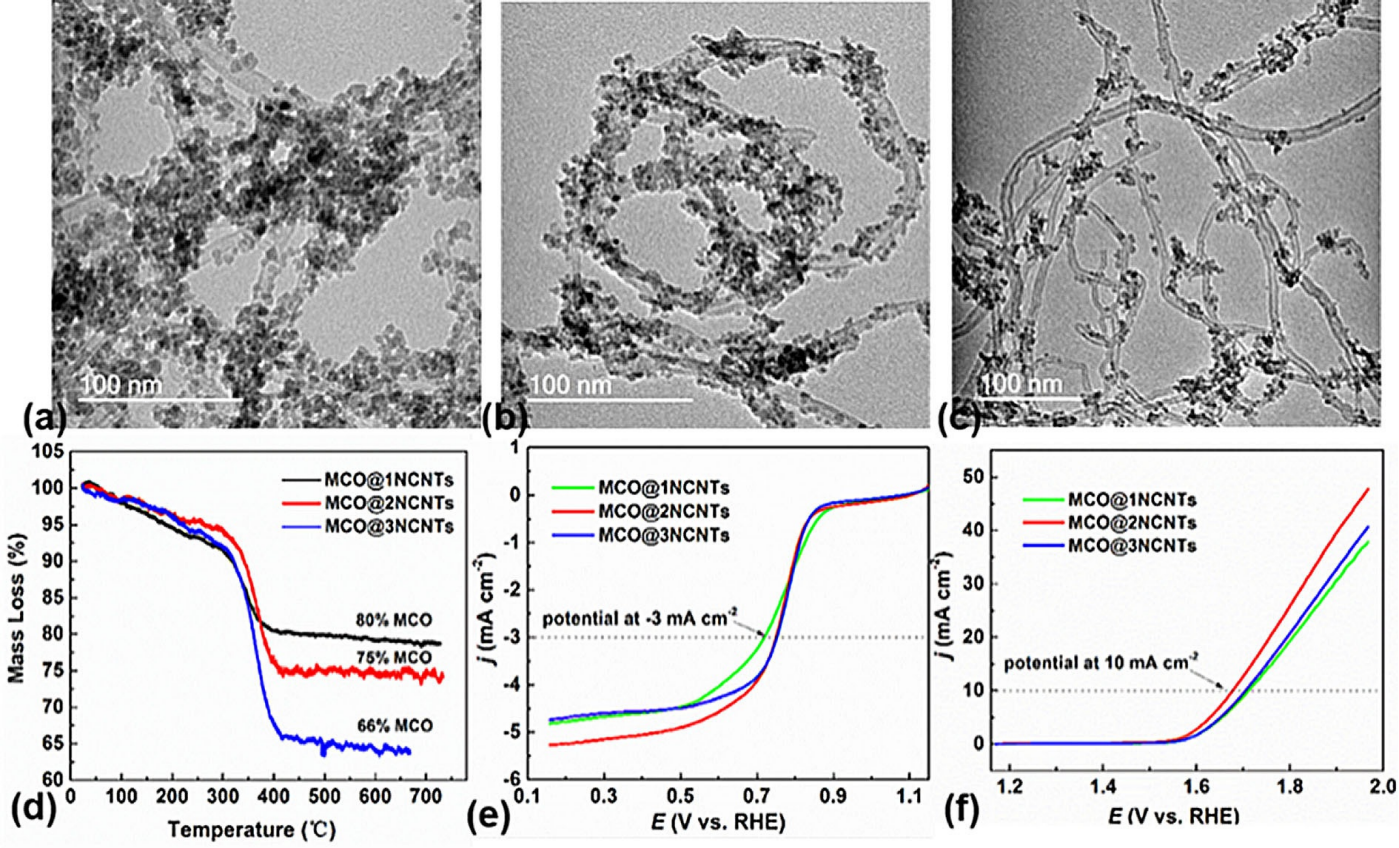
TEM images of a) MCO@1NCNTs, b) MCO@2NCNTs, and c) MCO@3NCNTs. d) Thermogravimetric (TG) curves of MCO@NCNTs recorded at a constant heating rate of 3 °C min^−1^ under a flow of air. The reported mass loss is after baseline correction measured against an empty crucible. e) ORR and f) OER LSV curves of MCO@1NCNTs, MCO@2NCNTs, and MCO@3NCNTs on a glassy carbon electrode measured in O_2_‐saturated 0.1 m KOH, at a scan rate of 10 mV s^−1^ and rotating speed of 1600 rpm.

Furthermore, we also show that the mass loading and surface functionalization of CNTs have a profound influence on the catalytic activity of the hybrids. For this purpose, we first investigated the effect of concentration of active MCO on the CNTs support (samples with increased CNT loadings were named MCO@1NCNTs, MCO@2NCNTs, and MCO@3NCNTs). The MCO nanocrystals in all samples are of cubic phase with a similar particle size of 3–5 nm (Figure 5 a–c and Figure S11 in the Supporting Information). However, a volcano‐type catalytic performance can be seen for both the ORR and OER, upon increasing the MCO loading from about 66 to 80 wt % (Figure 5 d–f and Figures S12 and S13 in the Supporting Information). The best catalytic performance is observed for MCO@2NCNTs with a MCO mass loading of 75 wt % in the hybrid (3:1 weight ratio of MCO/NCNTs, which is an atomic ratio of about 3:20 of MCO and NCNTs). As evidenced in the TEM images, the MCO@1NCNTs show an insufficient level of active MCO at the NCNTs, thus leading to somewhat less favorable ORR/OER activities. Notably, the high loading of MCO at NCNTs tends to agglomerate, which can smear out specific active surface area/sites, and also inhibits charge transport in the hybrids. Clearly, the MCO@2NCNTs hybrid exhibits a more balanced composition and evenly distributed nanoparticles, which synergistically enhances the overall catalytic activities. It is worth noting that the bifunctional performance of MCO@2NCNTs is comparable to that of some of the best bifunctional electrocatalysts reported in the literature (see Tables S1 and S2 in the Supporting Information).

Finally, surface oxidization of the CNTs may play a role in the catalytic performance of the hybrid. Metal oxide nanoparticles cannot be directly anchored on pure graphitic structures in the hydrophobic state, without surface oxygen groups, such as −COO and C−O.^40^, ^53^ On the other hand, excessive oxidation of CNTs is also detrimental to charge transport, specifically due to the alteration of structural integrity and reduced conductivity, with extensive transformation of sp^2^‐C to defective sp^3^‐C.^54^, ^55^

**Figure 6:**
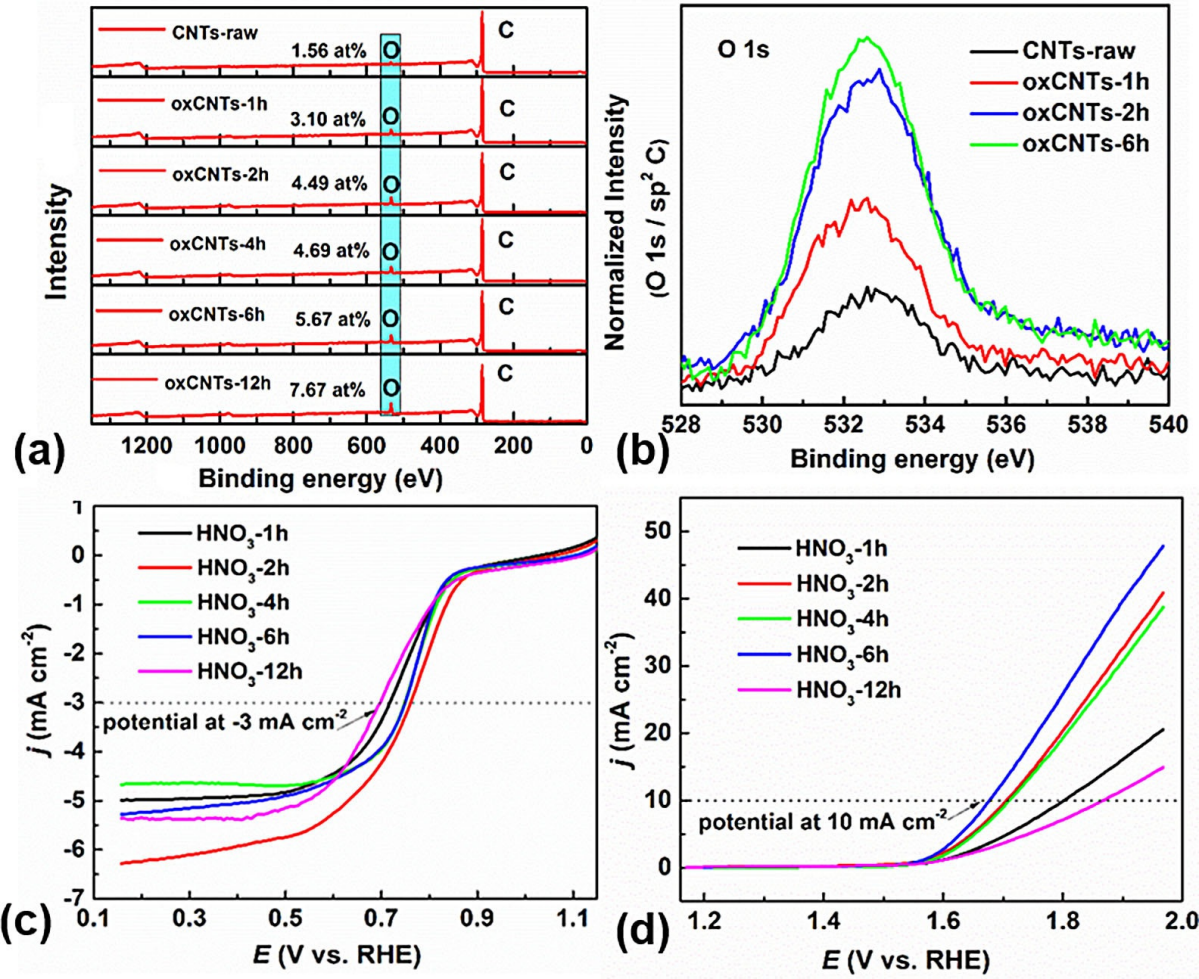
a) XPS surveys of oxCNTs‐*x* h; oxygen content in at %. b) Normalized intensity of O 1s to sp^2^‐C of representative samples. c) ORR and d) OER LSV curves of HNO_3_‐*x* h (*x=*1, 2, 4, 6, and 12) on a glassy carbon electrode measured in O_2_‐saturated 0.1 m KOH, at a scan rate of 10 mV s^−1^ and rotating speed of 1600 rpm.

By controlling the synthetic conditions, we modified the CNTs with an increased degree of oxidation. For instance, XPS estimated an increased oxygen content from 1.56 to 7.67 at % and the normalized intensity of O 1s increased with the degree of oxidation (Figure 6 a and b and Figure S14 in the Supporting Information). Accordingly, the clear influence of surface functionalization is seen in producing the hybrid structures, MCO@NCNTs, in which the CNTs treated for 1 h (HNO_3_‐1 h) exhibit a relatively small amount of MCO growth. Similarly, the highly oxidized CNTs (HNO_3_‐12 h) show a large amount of MCO nanoparticle growth and agglomeration, probably at large defect sites of missing carbons, under similar solvothermal and precursor concentration conditions (PXRD and TEM). Therefore, without a doubt, CNTs at a controlled degree of oxidation (HNO_3_‐2 h and HNO_3_‐6 h) to achieve an optimum mass loading of MCO lead to the best activity for the ORR and OER, respectively (Figure 6 b and c, and Figures S15–S17 in the Supporting Information).

Overall, our study reveals that highly exposed active sites and the strong interaction between MCO and CNTs are beneficial for electrocatalysis. Oxide nanoparticles act as catalytically active centers and CNTs play a critical role in enhancing electron conduction/charge transport by enabling shorter migration of charges and also providing a high surface area for the reactants. Clearly, MCO and NCNTs alone show limited electrocatalytic activity for the OER due to inferior electron conductivity and lack of effective active sites. Furthermore, the extensive agglomeration of MCO nanoparticles smears out the active surface. Hence, the hybrid MCO@NCNTs lead to enhanced activity for catalysis.

Moreover, the particle sizes, compositions, and crystallographic phases of active Mn_*x*_Co_3−*x*_O_4_ may also be important for the overall catalytic activity. Electrocatalysis only occurs at the solid (catalysts)–liquid (electrolyte)–gas (O_2_) three‐phase boundary. The larger size of the particles at CNTs leads to a decreased contact area of both catalysts and electrolyte. Additionally, Mn_1.5_Co_1.5_O_4_@NCNTs and Mn_2_CoO_4_@NCNTs exist in the form of the (Co, Mn) (Co, Mn)_2_O_4_ tetragonal phase. The cubic spinel MnCo_2_O_4_@NCNTs show better ORR activity than that of the tetragonal phase composites.^2^, ^25^, ^36^ Thus, our results further confirm that the cubic spinels of nanocrystals at partial manganese substitution for cobalt, MnCo_2_O_4_@NCNTs, show the best catalytic activity for the ORR, over that of the tetragonal spinels formed at relatively high manganese concentrations. The overall performance is also compromised by the respective composition of the MCO and CNTs, for which a high concentration of each component shows a detrimental effect on the activity. Finally, we also note that the surface oxidation of CNTs, to produce the efficient anchoring of MCO particles and surface reactive sites, is important in achieving favorable ORR and OER, or bifunctional, activity.

## Conclusions

The oxygen electrocatalytic performance of the manganese cobalt oxide/nitrogen‐doped multiwalled carbon nanotube hybrids (Mn_*x*_Co_3−*x*_O_4_@NCNTs) system is highly dependent on the synthetic methods, compositions, sizes, and phases of Mn_*x*_Co_3−*x*_O_4_, as well as the degree of oxidation of CNTs. Such activities can be further tuned by the mass loading of the MCO for both the oxygen reduction reaction (ORR) and oxygen evolution reaction (OER). The facile solvothermal synthetic method can lead to the formation of finely dispersed particles and strong coupling between metal oxide and carbon support. The level of Mn doping, or the Mn/Co ratio, in the spinels markedly influences the ORR and OER bifunctionality: a relatively low level of Mn doping enriches 3^+^ cations and redox pairs, favoring the ORR/OER; however, further increasing the level of Mn increases the particle size and favors a tetragonal phase, both of which are unfavorable for the ORR and OER (both activity and stability). Preoxidation of the CNTs in HNO_3_ is necessary to create defective sites for nucleation and anchoring of oxide particles, but excessive oxidation reduces the electron conductivity and structural integrity of the nanotubes. The optimum catalytic activities for the ORR and bifunctionality are obtained in the hybrid structure, MnCo_2_O_4_@NCNTs, at a mass ratio of oxides/CNTs of 3:1 (equivalent to about 3:20 atomic ratio) and a surface oxygen content of 4.5–5.5 at % in the CNTs, with nitrogen doping at 1.4 at %. Such hybrid structures show equivalent ORR and OER catalytic activities to that of the benchmark platinum and iridium standards, respectively. Moreover, the hybrid structures are bifunctional and highly stable over long‐term operations, and can be readily scaled up for alkaline fuel cells and metal–air battery applications.

## Experimental Section

### Chemicals

The following chemicals were used as received: multiwalled CNTs (≥98 %, Sigma–Aldrich), nitric acid (69 %, BDH), cobalt(II) acetate tetrahydrate (≥99 %, Sigma–Aldrich), manganese(II) acetate tetrahydrate (≥99 % Sigma–Aldrich), ethanol (96 %, VWR), solution of ammonia (35 %, Fisher Scientific), Nafion (5 wt % in alcohol and water, Sigma–Aldrich), and 20 % Pt/C (Alfa Aesar).

### Preoxidation of CNTs

The CNTs (300 mg) were oxidized in nitric acid (6 m, 25 mL) by heating at reflux (110 °C) for different periods of time (1, 2, 4, 6, and 12 h). Then, the reaction was cooled to room temperature and the precipitates were collected by centrifugation and washed with deionized (DI) water several times until pH 7. The precipitates were freeze‐dried to obtain the final products, oxCNTs‐*x* h (*x=*1, 2, 4, 6, and 12).

### Synthesis of MnCo_2_O_4_ nanoparticles anchored at NCNTs

In a typical reaction, oxCNTs‐2 h (12 mg) were dispersed in ethanol (24 mL) in a round‐bottomed flask and sonicated for several minutes to ensure uniform dispersion. Next, 0.6 m aqueous solutions of Co(OAc)₂**⋅**4 H_2_O and Mn(OAc)₂**⋅**4 H_2_O, were prepared in a 2:1 ratio (*v*/*v* 533 μL/267 μL) and added to the suspension of oxCNTs in ethanol. After stirring for 30 min, NH_3_ **⋅**H_2_O (500 μL) and DI water (300 μL) were added. The solution was then heated to 80 °C and left for 20 h under stirring before being cooled to room temperature. Finally, the reaction mixture was transferred to a 100 mL Teflon‐lined autoclave for solvothermal reaction at 150 °C for 3 h. The product was collected by centrifugation, washed with DI water several times, and freeze‐dried to obtain the final product, MCO@NCNTs.

Samples with different amounts of oxCNTs‐6 h were synthesized by adjusting the amount of oxCNTs‐6 h to 6, 12, and 18 mg, and denoted as MCO@1NCNTs, MCO@2NCNTs, and MCO@3NCNTs, respectively. Hybrids synthesized with different degrees of oxidation of oxCNTs‐*x* h in a similar manner contained 12 mg of oxCNTs and were denoted as HNO_3_‐*x* h (*x=*1, 2, 4, 6, and 12, which was the number of hours of oxidation).

Mn_*x*_Co_3−*x*_O_4_@NCNTs (0≤*x*≤3) hybrids were prepared by the same method with different mass ratios of metal salt precursors. Controlled MCO nanoparticles without NCNTs and NCNTs without oxide particles were also synthesized for comparison by using the same procedure. The physical mixture sample was made by mixing the MCO nanoparticles and NCNTs in an equivalent mass ratio to that of constituents in the hybrid MCO@NCNTs.

### Characterization

The morphology and elemental analysis of as‐prepared products were characterized by means of TEM (JEOL JEM‐2100F) and EDS (Oxford Instruments X‐Max^N^). The phase and structure was identified by PXRD (STOE StadiP) by using Mo_Kα_ radiation (*λ*=0.71 Å). The elemental composition was estimated by XPS (Thermo Scientific K‐alpha). The accurate mass ratio of MCO/CNTs in the hybrids was determined by TG (SETARAM Setsys 16/18) after complete burning of the CNTs under an oxidizing atmosphere, air. XPS fitting and analysis of all elements were carried out with CasaXPS software. All of the spectra were calibrated with the C 1s peak at 284.5 eV.

### Electrochemical measurements

The as‐synthesized catalyst (2 mg) and Nafion solution (18 μL, ≈40 wt % Nafion to catalyst ratio) were dispersed in DI water (482 μL) and sonicated in an ice–water bath for 1 h to form a uniform ink. Pt/C ink was made by means of the same method as a reference standard. The glassy carbon electrode (3 mm in diameter, Metrohm) was polished with Al_2_O_3_ powder first to obtain a mirror‐like surface and sonicated in DI water to remove any contaminants from the surface. Then the catalyst ink (5 μL, containing 20 μg of catalyst) was pipetted and loaded onto the surface of the prepolished glassy carbon electrode (loading of catalyst: 0.28 mg cm^−2^). The electrode was then dried at 55 °C in an oven. All electrochemical measurements were conducted on Autolab (Metrohm PGSTAT302N) in a three‐electrode electrochemical cell by using a platinum sheet (Metrohm), Ag/AgCl (saturated KCl, Sigma–Aldrich), and the catalyst‐modified glassy carbon as counter, reference, and working electrodes, respectively. The alkaline (0.1 m KOH, pH 13) electrolyte was purged with O_2_ in advance for 40 min to ensure that it was saturated with O_2_. The potentials were converted with respect to the RHE, *E*_RHE_=*E*_Ag/AgCl_+0.059×pH+0.197 V.

The ORR performance was first investigated by CV in N_2_‐ and O_2_‐saturated 0.1 m KOH at room temperature, with a scan rate of 10 mV s^−1^ in the potential range of 1.2 to 0.2 V versus the RHE. The working electrode was subjected to repeated CV runs until a stable current response was obtained. The actual CV data was then recorded at a scan rate of 10 mV s^−1^ in an O_2_‐saturated electrolyte. In control experiments, CV measurements were also performed in a N_2_‐saturated electrolyte, which normally did not show the oxygen reduction cathodic current peak. LSV of a RDE was conducted at different speeds between 400 to 2000 rpm, at a scan rate of 10 mV s^−1^.

The ratio of generated intermediate species and the number of transferred electrons (*n*) can be calculated from the results of a RRDE (Pt‐ring/glassy carbon disk, with a diameter of 5 mm for disk, Metrohm). The working electrode was prepared by the same method as that used for the RDE. The disk electrode was scanned cathodically at a rate of 10 mV s^−1^, and the ring potential was maintained at 1.46 V versus RHE to oxidize the intermediate species completely. The ratio of generated HO_2_^−^ and the electron‐transfer number could be calculated by using Equations (1), (2), respectively:^56^

(1) $$\%\left(HO_{2}^{-}\right)=200\times \frac{I_{r}/N}{I_{d}+I_{r}/N}$$

(2) $$n=4\times \frac{I_{d}}{I_{d}+I_{r}/N}$$

in which *I*_d_ is the disk current, *I*_r_ is the ring current, and *N* is the current collection efficiency of the Pt ring. Here, *N*=0.25.

For the Tafel plot calculation, for the ORR, the kinetic current was calculated from the mass‐transport correction of RDE (1600 rpm) by using Equation (3):

(3) $$j_{k}=\frac{j\times j_{L}}{\left(j_{L}-j\right)}$$

By plotting potential against log *j*_k_ from LSV curves, the ORR Tafel slopes can be obtained.

The OER Tafel slopes can be obtained directly by plotting potential against log *j* from LSV curves.

All current densities were normalized to the geometric area of the electrode (the disk diameters are 3 and 5 mm for the RDE and RRDE, respectively).

CA tests for the ORR were conducted at 0.66 V versus RHE and 1600 rpm for 15 h. OER tests were investigated by the LSV method in the potential region of 1.2 to 2.0 V versus RHE at a sweep rate of 10 mV s^−1^ in 0.1 m KOH under a rotating speed of 1600 rpm. These tests were normally conducted after the CV and ORR LSV tests. The stability for oxygen evolution was also evaluated with CA at 1.66 V versus RHE and 2500 rpm for 3 h. All of our tests were carried out in 0.1 m KOH without *IR* compensation.

XANES measurements were carried out to study the effects of valence change in Mn_*x*_Co_3−*x*_O_4_@NCNTs. Experimental details can be found in the Supporting Information.

## Conflict of interest

*The authors declare no conflict of interest*.

## Supporting information

As a service to our authors and readers, this journal provides supporting information supplied by the authors. Such materials are peer reviewed and may be re‐organized for online delivery, but are not copy‐edited or typeset. Technical support issues arising from supporting information (other than missing files) should be addressed to the authors.

Supplementary
