# Supplementary material for: Tunable Bifunctional Activity of MnxCo3−xO4 Nanocrystals Decorated on Carbon Nanotubes for Oxygen Electrocatalysis
Source: ChemSusChem. 2018 Mar 26;11(8):1295–304. doi: 10.1002/cssc.201800049 (PMC5947553; doi:10.1002/cssc.201800049)
Supplement: Supplementary file 1 — Supplementary [file CSSC-11-1295-s001.pdf]

CHEMISTRY & SUSTAINABILITY
CHEMSUSCHEM
ENERGY & MATERIALS

# Supporting Information

# Tunable Bifunctional Activity of $Mn_xCo_{3-x}O_4$ Nanocrystals Decorated on Carbon Nanotubes for Oxygen Electrocatalysis

Tingting Zhao,[a] Srinivas Gadipelli,[a] Guanjie He,[a] Matthew J. Ward,[b] David Do,[c] Peng Zhang,[c] and Zhengxiao Guo*[a]

cssc_201800049_sm_miscellaneous_information.pdf

**Experimental Methods**

**X-ray Absorption Near Edge Structure (XANES) measurements**

XANES measurements were conducted at the bending magnet-based 20BM beamline of the Advanced Photon Source (APS) at Argonne National Laboratory. The APS is a third generation 7.0 GeV synchrotron light source providing a constant 102 mA of current (24 singlets) operating in top-up mode. The 20BM beamline of the APS uses a Si (111) double crystal monochromator for energy selection and a Rh-coated mirror for higher order harmonic rejection, and provides a flux of ~$10^{11}$ photons/s @ 10 keV. All samples were prepared by calculating the mass of sample required to give a theoretical total absorption of less than 2 absorption lengths, mixing with approximately 100 mg of boron nitride powder (Alfa Aesar 99.5% metals basis), and grinding thoroughly in an agate mortar and pestle for >20 mins. The powder was then pressed into 13 mm diameter pellets, and the resultant pellets were sealed in a single layer of 1 Mil Kapton tape. XANES at the manganese and cobalt K-edges, at *ca.* 6538 eV and 7709 eV respectively, were measured in transmission geometry at 90° to the X-ray beam using ion chambers upstream ($I_0$) and downstream ($I_t$) of the sample. A manganese (cobalt) reference foil was also measured simultaneously for energy calibration downstream of the sample between $I_t$ and a reference ion chamber ($I_{ref}$) placed downstream of the foil. A 50/50 mixture of He and $N_2$ gasses was used to fill the $I_0$ ion chamber and 100% $N_2$ gas was used to fill $I_t$ and $I_{ref}$ ion chambers. The monochromator was tuned to full intensity *ca.* 300 eV above the absorption edge (Mn or Co) and then detuned 15% from the maximum. The harmonic rejection mirror was set at an angle of 7 mrad giving a high energy cut off of ~ 9.57 keV. The energy steps for Mn XANES scans were set to 5 eV in the pre-edge region ($E_0$-200 eV to $E_0$-30 eV), 0.25 eV in the XANES region ($E_0$-30 eV to $E_0$+30 eV), and 0.05 Å$^{-1}$ in the Extended X-ray Absorption Fine Structure (EXAFS) region ($E_0$+30 eV to $E_0$+566 eV). For Co XANES scans the same step sizes and energy ranges were

selected for the pre-edge and XANES regions, but the EXAFS region was scanned in 0.05 $Å^{-1}$ steps from $E_0$+30 eV to $E_0$+973 eV. The total absorption was measured at the experimentally determined white-line (most intense feature in the XANES) position for all samples at both Mn and Co K-edges and was determined to be less than 2 absorption lengths for all samples. A blank pellet (100 mg BN powder) was also measured at both Mn and Co K-edges. The blank was found to contain no measurable Co or Mn contamination.

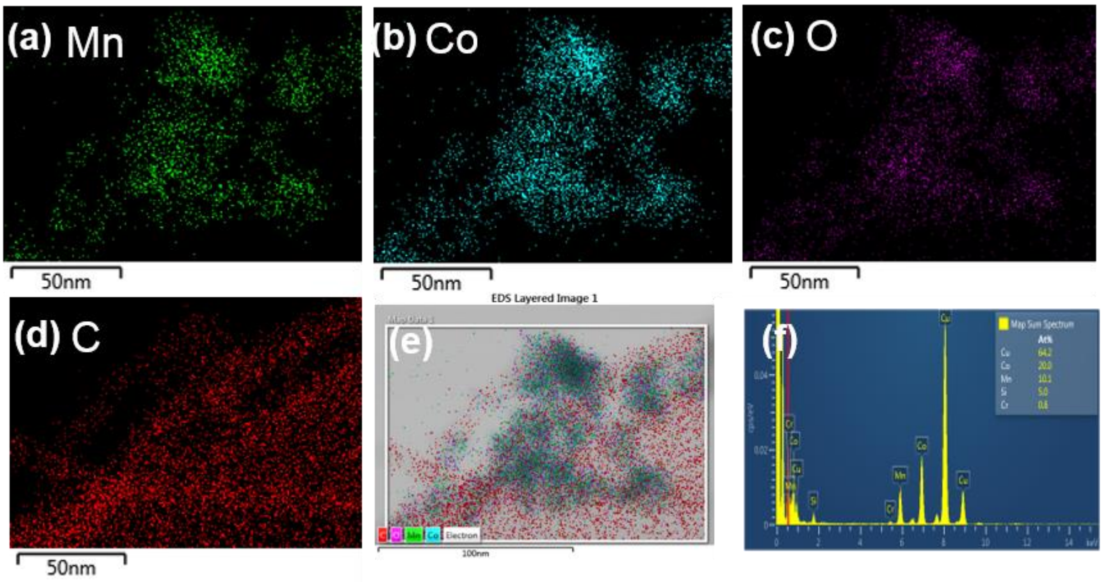


**Figure S1.** Elemental mapping images of a typical MCO@NCNTs hybrid: (a) Mn, (b) Co, (c) O, (d) C, (e) EDS layered image, and (f) EDS data for MCO@NCNTs.

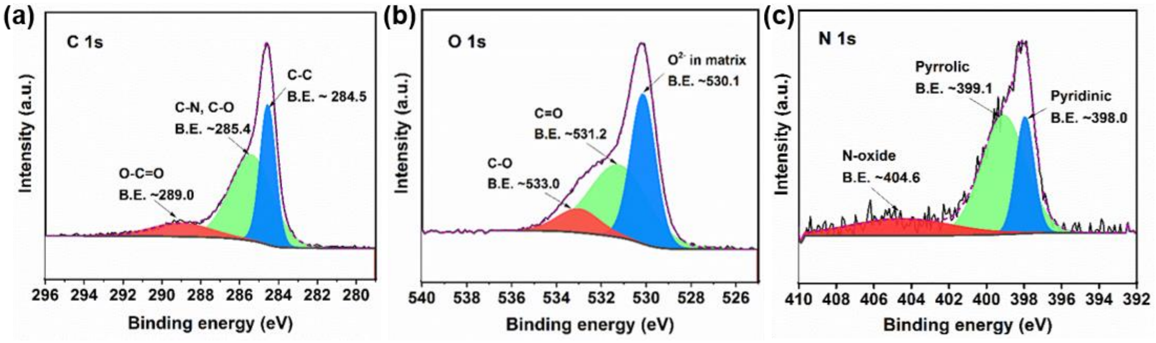


**Figure S2.** XPS analysis of MCO@NCNTs. Core level spectra of (a) C 1s, (b) O 1s and (c) N 1s.

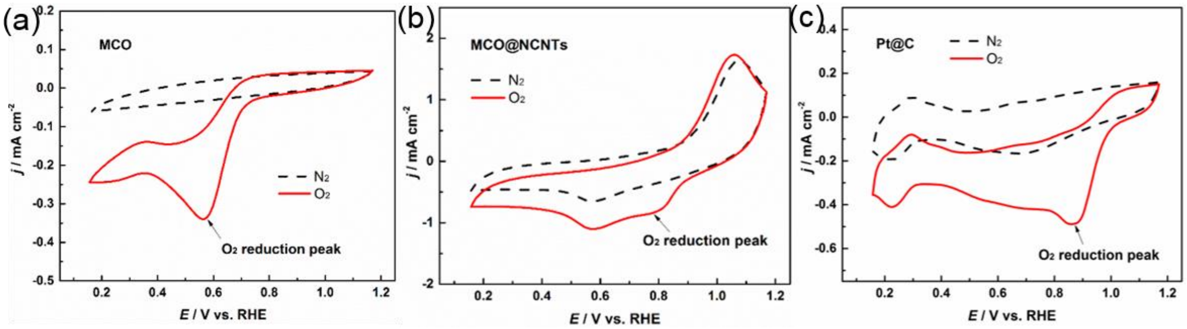


**Figure S3.** CV curves of the samples on glassy carbon electrode in $O_2$-saturated and $N_2$-saturated 0.1 M KOH electrolyte (a) MCO, (b) MCO@NCNT and (c) Pt@C.

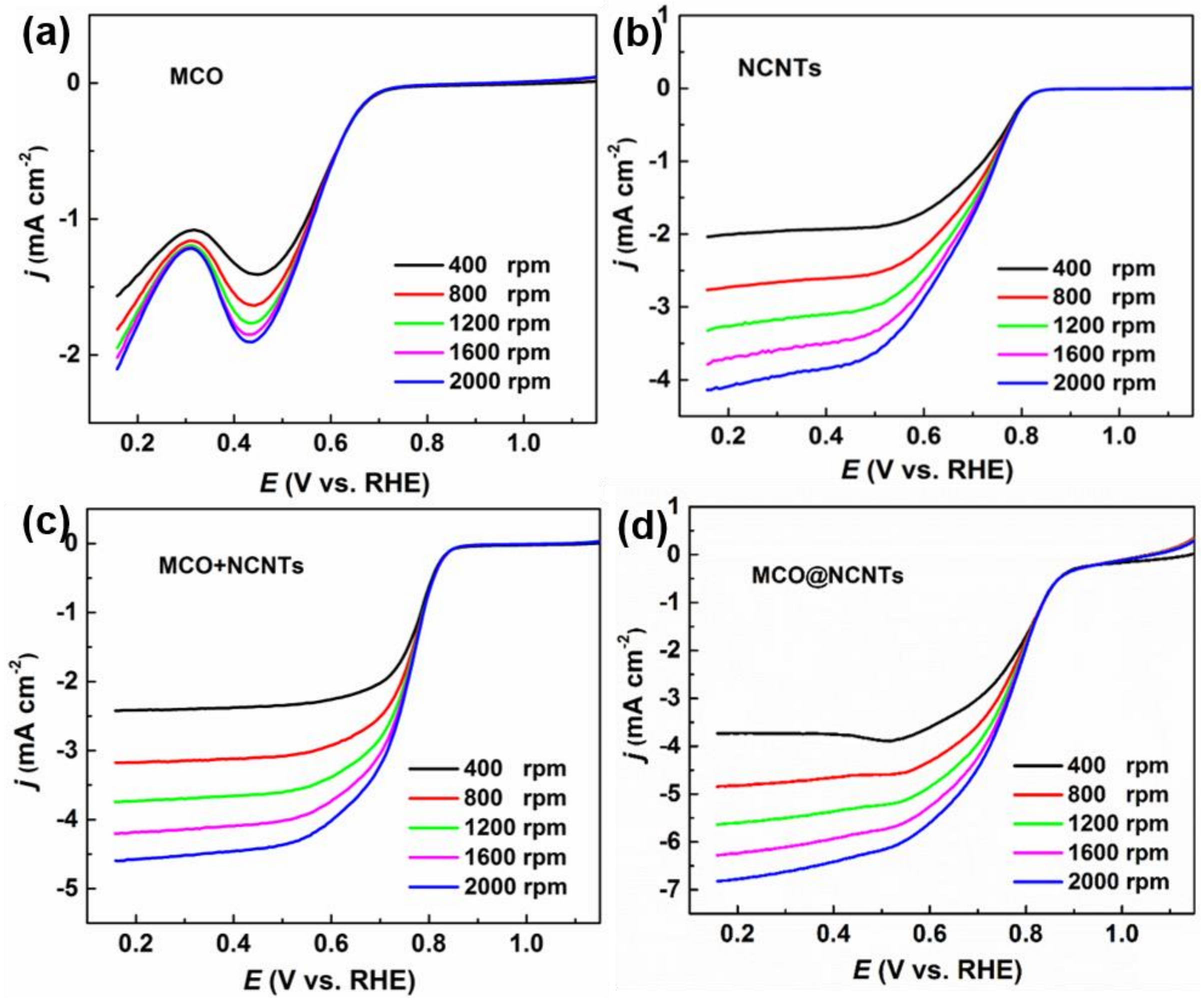


**Figure S4.** Rotating-disk liner sweep voltammograms of MCO, NCNTs, MCO+NCNTs and MCO@NCNTs in $O_2$-saturated 0.1 M KOH with a sweep rate of 10 mV/s at different rotation rates indicated.

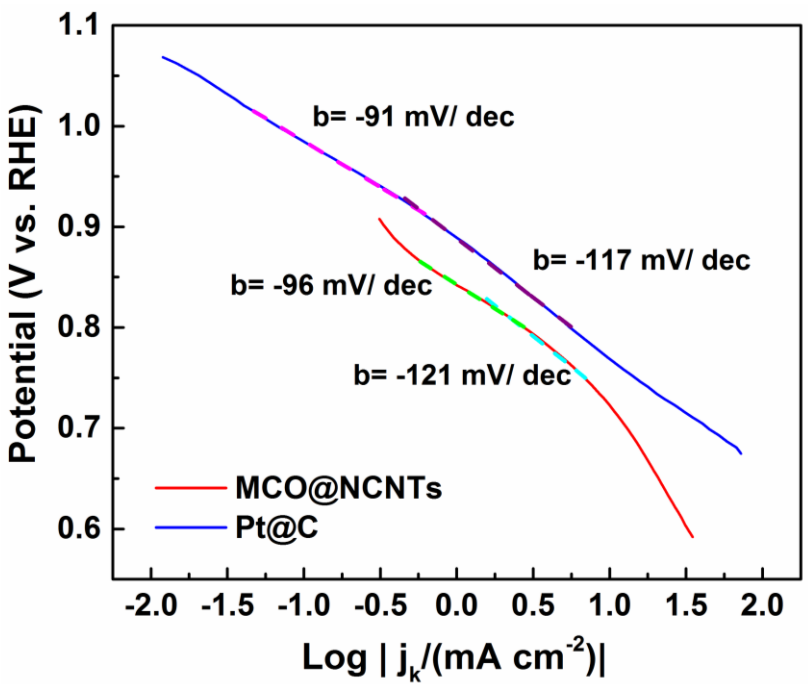


**Figure S5.** ORR Tafel plots of MCO@NCNTs and Pt@C derived by the mass-transport correction of corresponding RDE data (1600 rpm), without IR compensation.

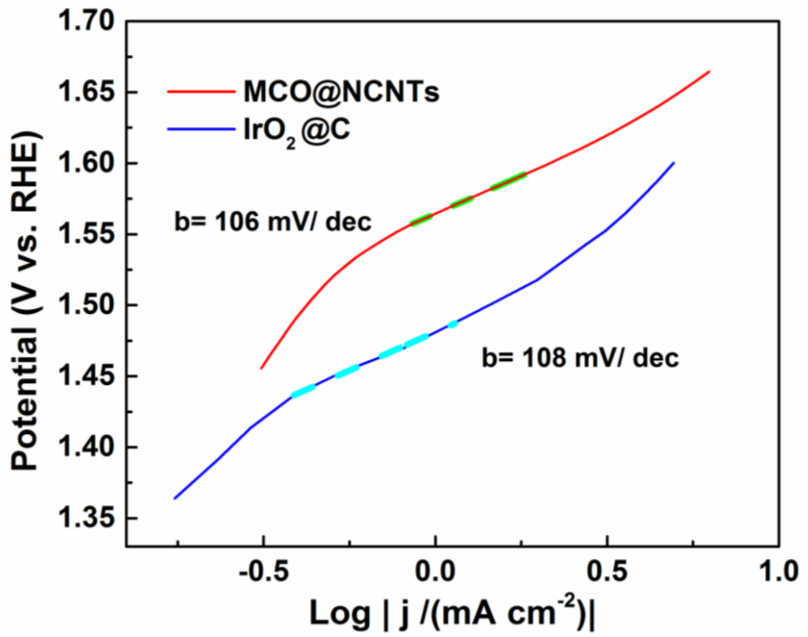


**Figure S6.** OER Tafel plots of MCO@NCNTs and $IrO_2$@C derived by corresponding RDE data (1600 rpm), without IR compensation.

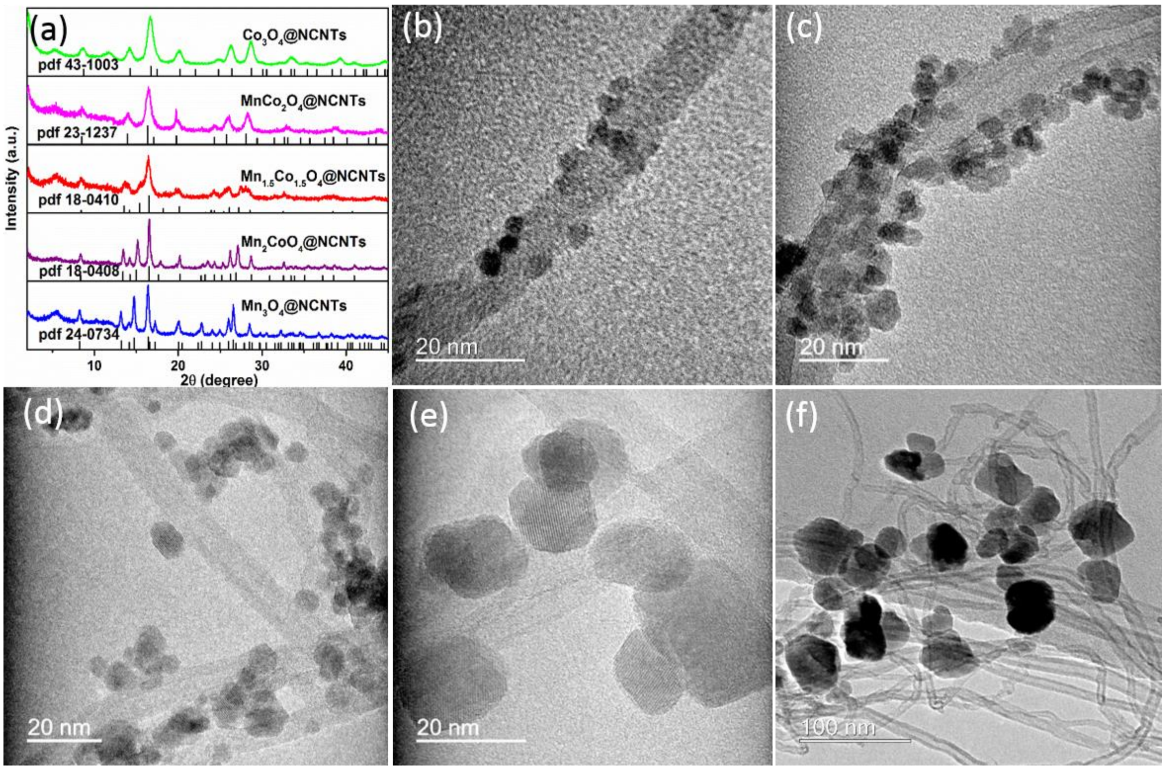


**Figure S7.** (a) PXRD patterns of MCO@NCNTs with different Mn/Co ratios and TEM images of (b) $Co_3O_4$@NCNTs, (c) $MnCo_2O_4$@NCNTs, (d) $Mn_{1.5}Co_{1.5}O_4$@NCNTs, (e) $Mn_2CoO_4$@NCNTs and (f) $Mn_3O_4$@NCNTs.

This increase in crystallite sizes with the increasing substitution of Mn is directly related to a reduced coordination effect of ammonia to Mn cations than to Co cations in the nucleation step, and hence reduced rate of effective nucleation.

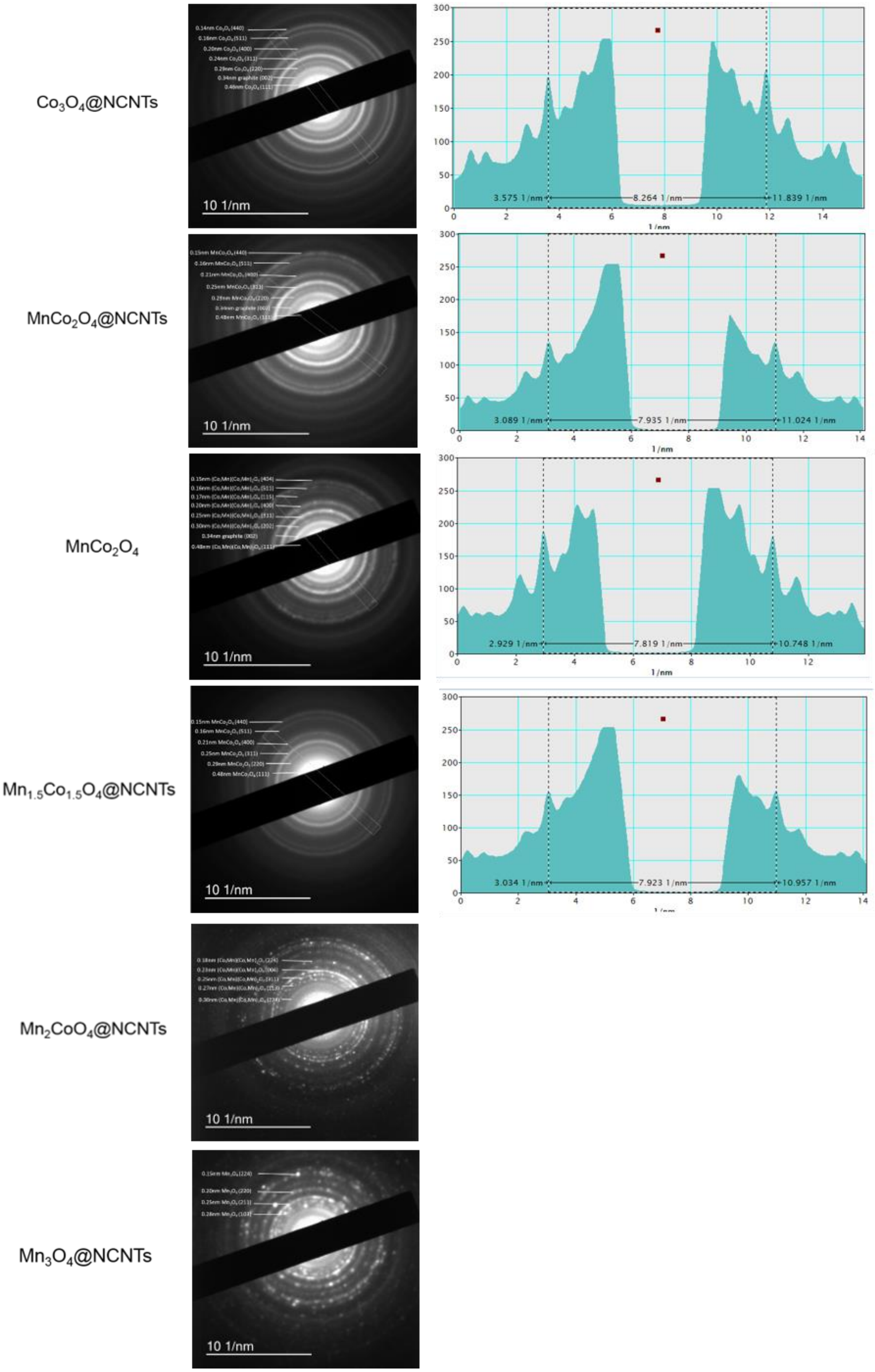


**Figure S8.** Electron diffraction patterns of $Mn_xCo_{3-x}O_4$@NCNTs (x=0, 1, 1.5, 2, 3) and $MnCo_2O_4$.

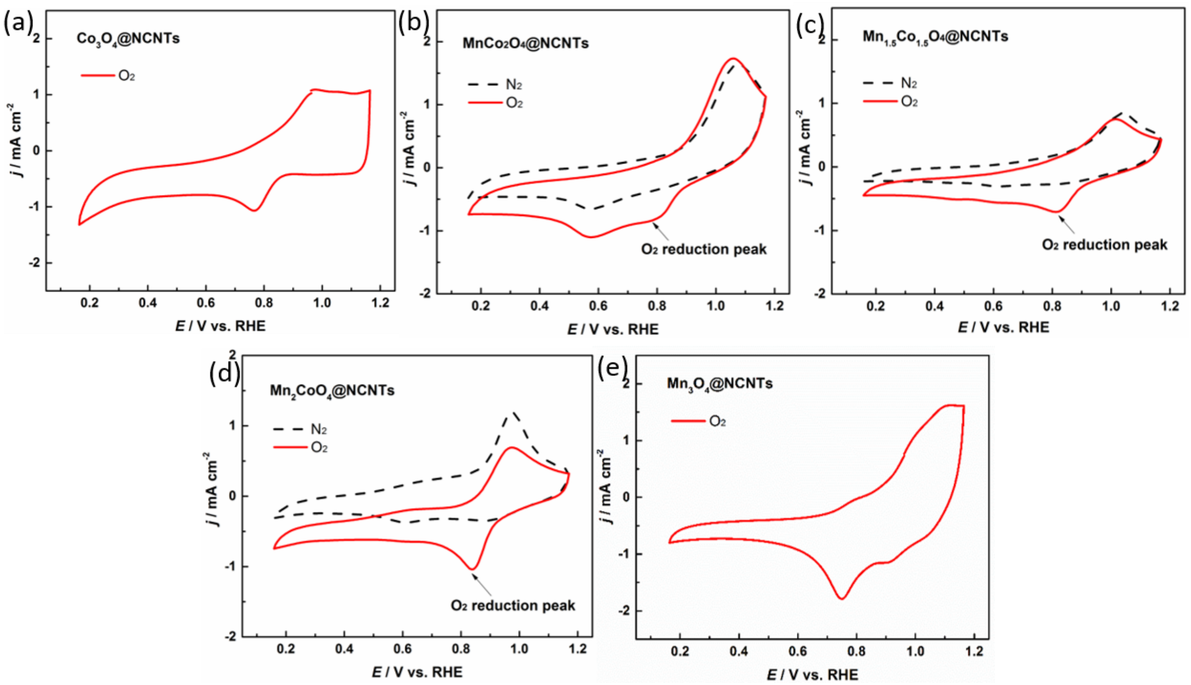


**Figure S9.** CV curves of the MCO@NCNT on glassy carbon electrode in $O_2$-saturated and $N_2$-saturated 0.1 M KOH electrolyte (a) $Co_3O_4$@NCNTs, (b) $MnCo_2O_4$@NCNTs, (c) $Mn_{1.5}Co_{1.5}O_4$@NCNTs, (d) $Mn_2CoO_4$@NCNTs and (e) $Mn_3O_4$@NCNTs.

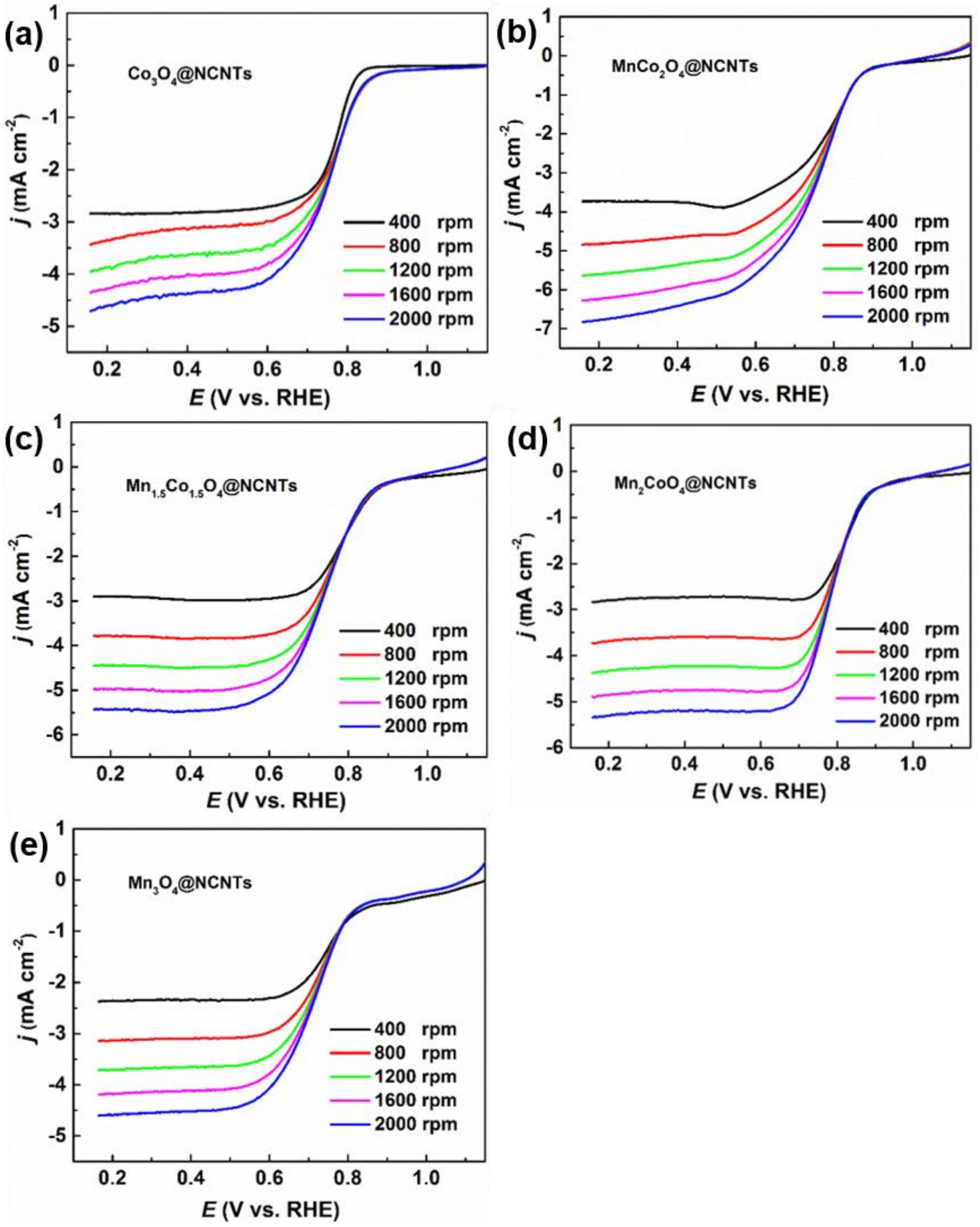


**Figure S10.** Rotating-disk liner sweep voltammograms of $Mn_xCo_{3-x}O_4$@NCNTs (x = 0, 1, 1.5, 2 and 3) in $O_2$-saturated 0.1 M KOH with a sweep rate of 10 mV/s at different rotation rates indicated.

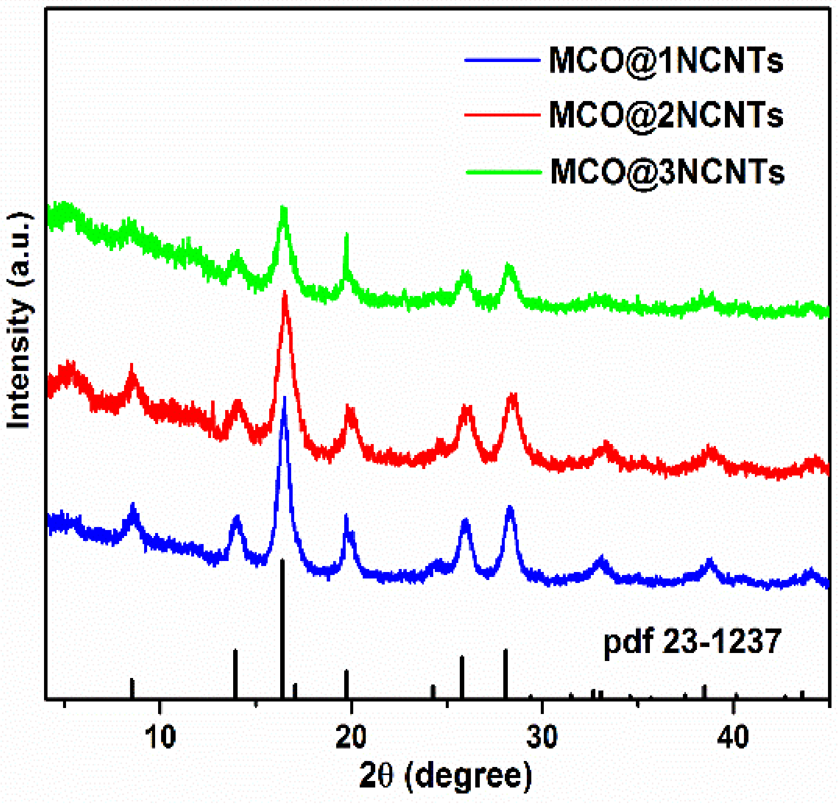


**Figure S11.** PXRD patterns of MCO@xNCNTs

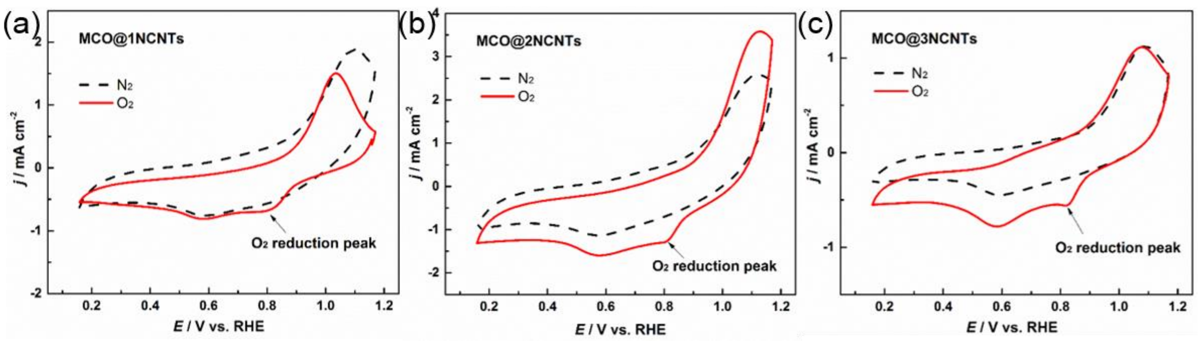


**Figure S12.** CV curves of the MCO@NCNTs on glassy carbon electrode in $O_2$- and $N_2$-saturated 0.1 M KOH electrolyte (a) MCO@1NCNTs, (b) MCO@2NCNTs, (c) MCO@3NCNTs.

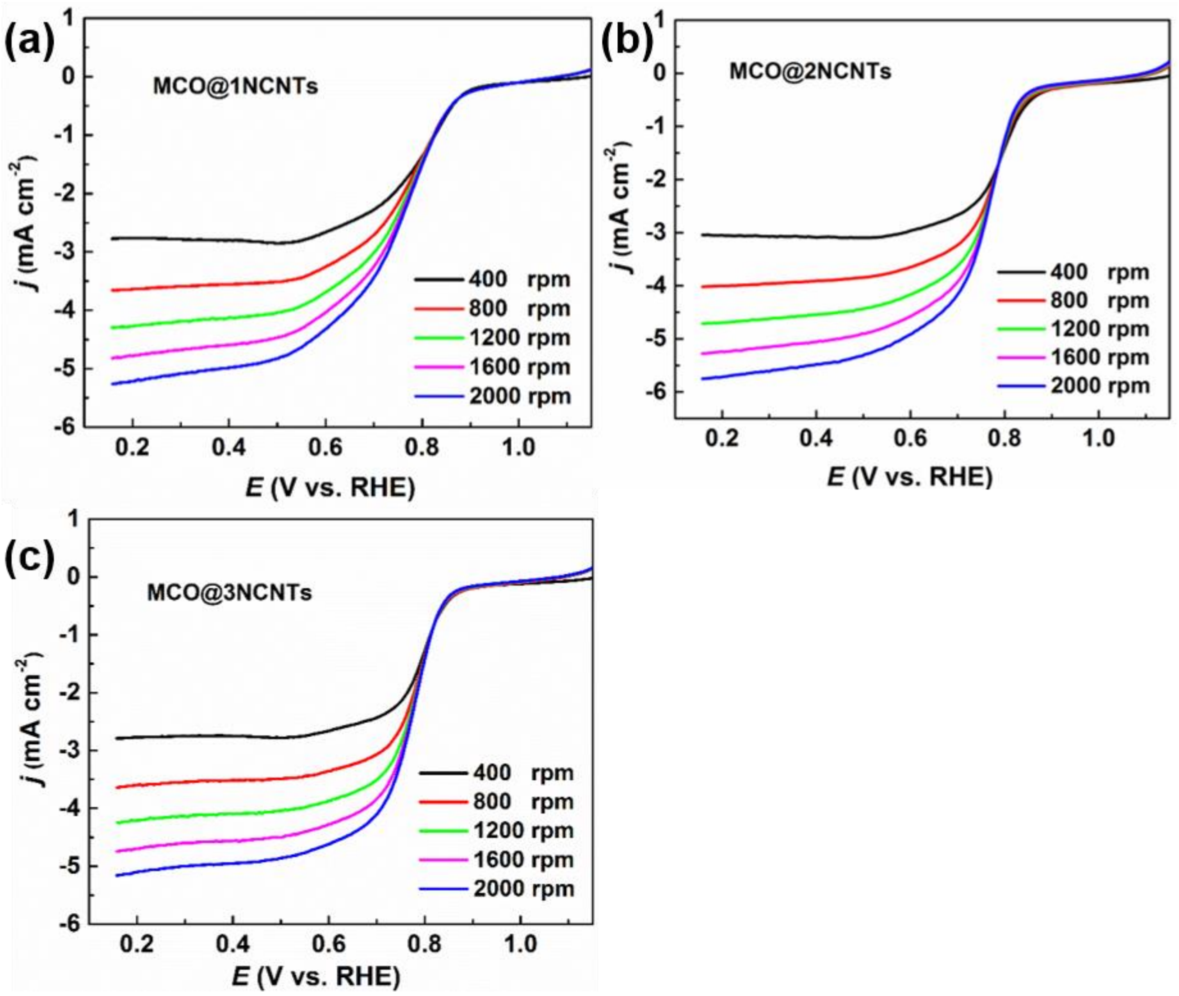


**Figure S13.** Rotating-disk liner sweep voltammograms of MCO@1NCNTs, MCO@2NCNTs and MCO@3NCNTs in $O_2$-saturated 0.1 M KOH with a sweep rate of 10 mV/s at different rotation rates indicated.

**Table S1.** Bifunctionality comparison of MCO@xNCNTs samples (x=1, 2, 3)

| Samples | ORR benchmark<br>E[V] at j = -3 mA $cm^{-2}$ | OER benchmark<br>E[V] at j = 10 mA $cm^{-2}$ | Bifunctional catalyst benchmark<br>$\Delta E$ = OER-ORR [V] |
|---|---|---|---|
| MCO@1NCNTs | 0.71 | 1.71 | 1.00 |
| MCO@2NCNTs | 0.74 | 1.67 | 0.93 |
| MCO@3NCNTs | 0.75 | 1.70 | 0.95 |

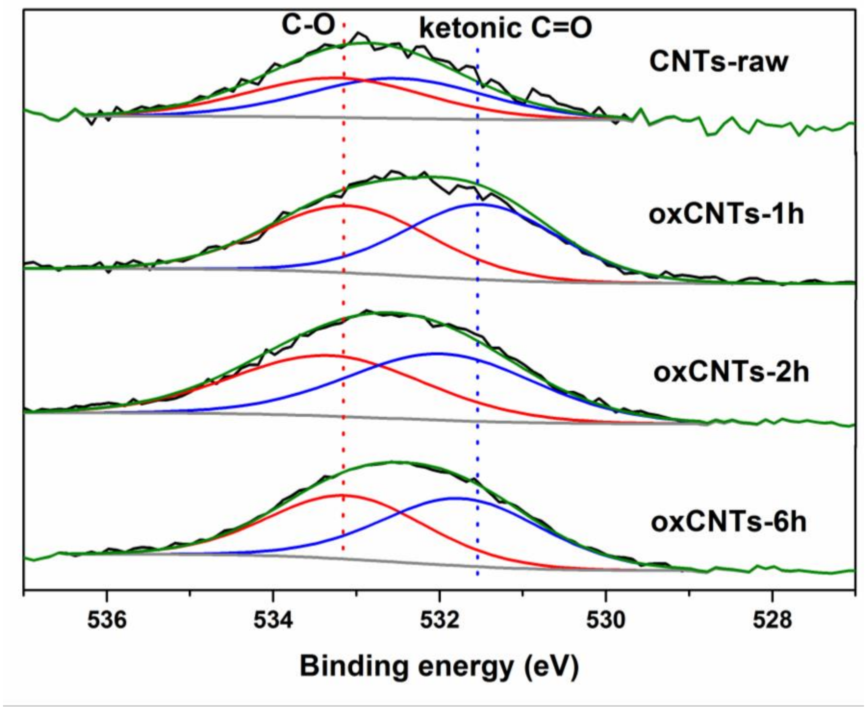


**Figure S14.** XPS core level spectra of CNTs-raw, oxCNTs-1h, oxCNTs-2h and oxCNTs-6h.

With increasing degree of oxidation of the sample, O 1s spectra shows a gradually increasing intensity, which means the increase of oxygen content. O 1s can be resolved to two peaks for the C-O (533.1$\pm$0.3 eV) and C=O 531.2 $\pm$ 0.3 eV), respectively. After oxidation, the peak for C=O shifts negatively, indicating a clear increase in the degree of oxidation. The quantitative analysis results of O 1s indicate an almost equal increase of both C-O and C=O.

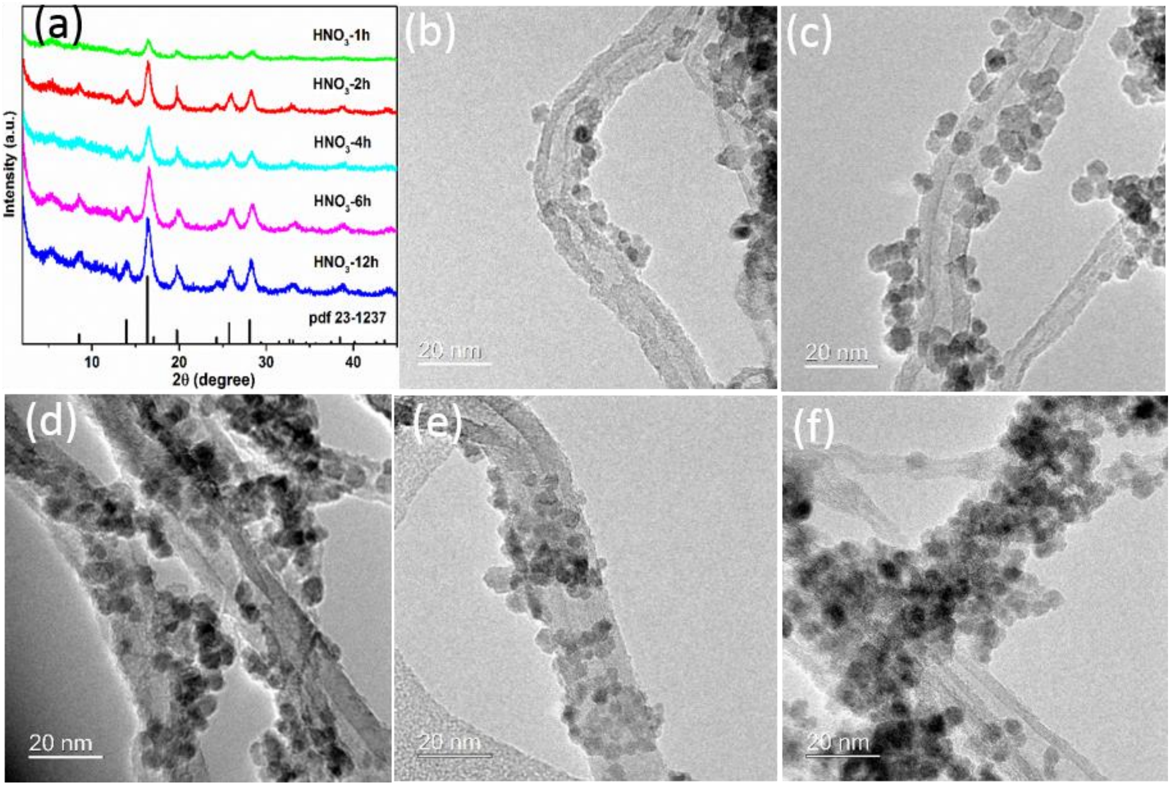


**Figure S15.** (a) PXRD patterns of $HNO_3$-xh samples and TEM images of the hybrids (a) $HNO_3$-1h, (b) $HNO_3$-2h, (c) $HNO_3$-4h, (d) $HNO_3$-6h and (e) $HNO_3$-12h.

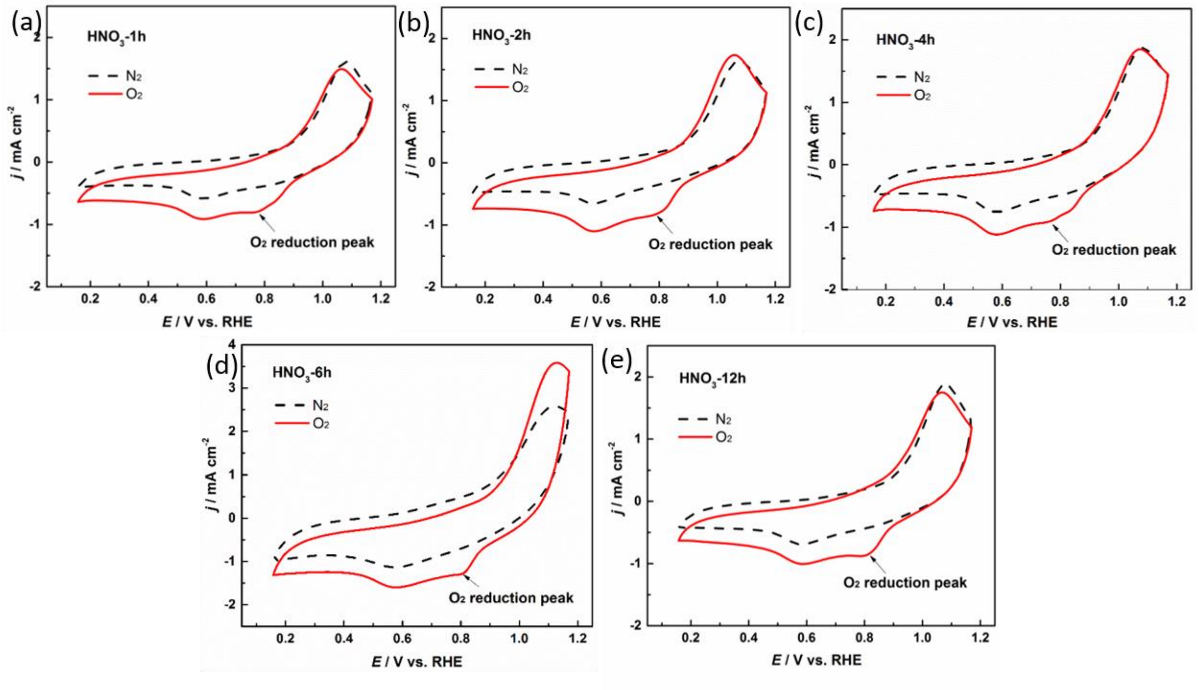


**Figure S16.** CV curves of the $HNO_3$-xh samples on glassy carbon electrodes in $O_2$-saturated or $N_2$-saturated 0.1 M KOH electrolyte (a) $HNO_3$-1h, (b) $HNO_3$-2h, (c) $HNO_3$-4h, (d) $HNO_3$-6h and (e) $HNO_3$-12h.

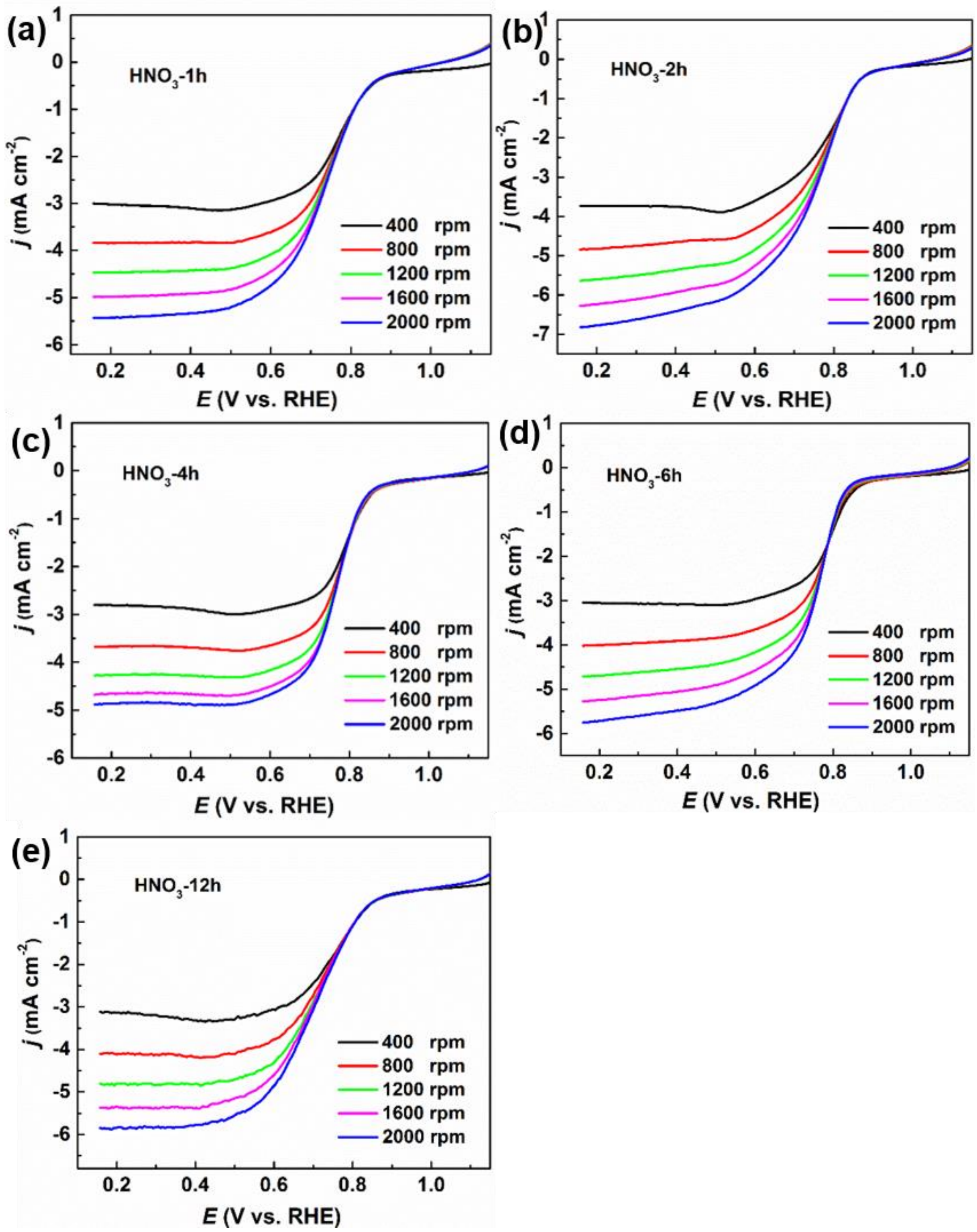


**Figure S17.** Rotating-disk liner sweep voltammograms of $HNO_3$-xh (x = 1, 2, 4, 6 and 12) in $O_2$-saturated 0.1 M KOH with a sweep rate of 10 mV/s at different rotation rates indicated.

**Table S2** ORR/OER activity comparison of bifunctional oxygen electrocatalysts

| No. | Samples | ORR | | OER | Bifuncti-onality | Mass loading (mg $cm^{-2}$) | Electrolyte | Ref. electrode | Ref. |
|---|---|---|---|---|---|---|---|---|---|
| | | $E_{onset}$ | $E_{-3}$ | $E_{10}$ | $\Delta E = E_{10}-E_{-3}$ [V] | | | | |
| 1 | Spinel Mn-Co Oxide in N-doped CNTs | 0.84 | — | 1.77 | — | 0.21 | 0.1 M KOH | RHE | [1] |
| 2 | CoMnO/C | 0.92 | 0.84 | 1.79 | 0.95 | 0.054 mg oxide $cm^{-2}$ | 0.1 M KOH | RHE | [2] |
| 3 | $MnCo_2O_4$/PPy | -0.08 | -0.23 | 0.79 | 1.02 | 0.4 | 0.1 M KOH | Ag/AgCl | [3] |
| 4 | Mesoporous $Co_3O_4$ | 0.70 | 0.60 | 1.64 | 1.04 | 0.1 | 0.1 M KOH | RHE | [4] |
| 5 | $Co_3O_4$/multi-walled carbon nanotube | -0.19 | -0.32 | 0.73 | 1.05 | — | 0.1 M KOH | Ag/AgCl | [5] |
| 6 | $Co_3O_4$/N-doped carbon nanoweb | -0.19 | -0.26 | 0.57 (2000 rpm) | 0.83 | — | For OER 1M KOH | SCE | [6] |
| 7 | Co/CoO nanoparticles on Co-N-doped carbon | -0.12 | -0.18 | 0.65 | 0.83 | — | 0.1 M KOH | Ag/AgCl | [7] |
| 8 | Co@$Co_3O_4$-NC1 | 0.87 | 0.79 | 1.66 | 0.87 | — | 0.1 M KOH | RHE | [8] |
| 9 | Cobalt carbonate hydroxide/C | 0.88 | 0.83 | 1.74 | 0.91 | 0.18 mg Co $cm^{-2}$ | 0.1 M KOH | RHE | [9] |
| 10 | $CoMn_2O_4$/ N-rGO | None | 0.78 | 1.68 | 0.90 | — | 0.1 M KOH | RHE | [10] |
| 11 | $MnCo_2O_4$ | 0.81 | 0.63 | 1.75 | 1.12 | For OER 0.051 | 0.1 M KOH | RHE | [11] |
| 12 | $CoMn_2O_4$ | 0.85 | 0.75 | 1.83 | 1.08 | — | | | |
| 13 | Non spinel NCNT/$Co_xMn_{1-x}O$ | 0.91 | 0.82 | 1.58 | 0.76 | 0.21 | 1 M KOH | RHE | [12] |
| 14 | dp-$MnCo_2O_4$/N-rGO | -0.07 | -0.15 | 0.77 | 0.92 | 0.1 | 0.1 M KOH | Ag/AgCl | [13] |
| 15 | dp-$MnCo_2O_4$/CNT | -0.10 | -0.23 | 0.70 | 0.93 | 0.1 | 0.1 M KOH | Ag/AgCl | |
| 16 | $Co_3O_4$/$Co_2MnO_4$ | 0.83 | 0.68 | 1.77 | 1.09 | 0.25 | 0.1 M KOH | RHE | [14] |
| 17 | $Mn_xO_y$/C | 0.82 | 0.70 | 1.67 | 0.97 | — | 0.1 M KOH | RHE | [15] |
| 18 | $Co_xO_y$/C | 0.81 | 0.71 | 1.66 | 0.95 | — | | | |
| 19 | $Ni_xO_y$/C | 0.75 | 0.59 | 1.64 | 1.05 | — | | | |
| 20 | Co/N-C | 0.18 | -0.64 | 0.68 | 1.32 | — | 0.1 M KOH | SCE | [16] |
| 21 | 3D crumpled graphene-CoO | — | — | 1.57 | — | — | 1 M KOH | RHE | [17] |
| 22 | $MnO_x$ | 0.82 | 0.72 | 1.61 | 0.89 | | 0.1 M KOH | RHE | [18] |
| 23 | $NiCo_2S_4$@graphene | -0.04 | -0.23 | 0.74 | 0.97 | 0.28 | 0.1 M KOH | Ag/AgCl | [19] |
| 24 | $NiCo_2O_4$-CNTs | 0.87 | 0.80 | 1.66 | 0.86 | 0.1 | 0.1 M KOH | RHE | [20] |
| 25 | $NiCo_2S_4$ sub- | -0.15 | -0.29 | — | — | — | 0.1 M KOH | Ag/AgCl | [21] |

| | | | | | | | | | |
|---|---|---|---|---|---|---|---|---|---|
| | micron spheres | | (1500 rpm) | | | | | | |
| 26 | $NiCo_2O_4$ | 0.86 | 0.78 | 1.63 | 0.85 | — | 0.1 M KOH | RHE | [22] |
| 27 | $NiCo_2O_4$ nanowire | -0.18 | -0.30 | 0.67 | 0.97 | — | 0.1 M KOH | Ag/AgCl | [23] |
| 28 | (Ni,Co)/CNT aerogel | 0.84 | 0.74 | 1.47 | 0.87 (0.73) | — | For ORR: 0.1 KOH For OER: 1 M KOH | — | [24] |
| 29 | $CoS_x$@N and S co-doped graphene nanosheets | -0.19 | — | 0.75 | — | — | 0.1 M KOH | Ag/AgCl | [25] |
| 30 | $Co_{0.5}Fe_{0.5}S$@N-mesporous graphitic | 0.86 | 0.80 | 1.65 | 0.85 | 0.8 | For ORR: 0.1 M KOH For OER: 1 M KOH | RHE | [26] |
| 31 | $CoFe_2O_4$ hollow nanospheres | 0.69 | 0.58 | 1.69 | 1.11 | 0.16 | 0.1M KOH | RHE | [27] |
| 32 | $CoFe_2O_4$/CNT | -0.2 | -0.28 | 0.73 | 1.01 | 1.006 | 0.1M KOH | Ag/AgCl | [28] |
| 33 | $CoFe_2O_4$/graphene | -0.17 | -0.24 | 0.74 | 0.98 | 1.006 | 0.1M KOH | Ag/AgCl | [29] |
| 34 | $FeCo_2O_4$/hollow graphene spheres | -0.14 | -0.21 | 0.76 | 0.97 | 1.006 | 0.1M KOH | Ag/AgCl | [30] |
| 35 | $CoFe_2O_4$/N,S-rGO | -0.15 | -0.23 | 0.74 | 0.97 | — | 0.1 M KOH | Ag/AgCl | [31] |
| 36 | $Ni_{0.5}Co_{0.5}Fe_2O_4$ hollow nanosphere | -0.25 | -0.34 | 0.73 | 1.07 | 0.4 | 0.1 M KOH | Ag/AgCl | [32] |
| 37 | $Ni_{0.5}Co_{0.5}Fe_2O_4$ hollow nanosphere | -0.30 | -0.38 | 0.66 | 1.04 | | | | |
| 38 | Copper doping in $Mn_2O_3$ | -0.16 | -0.31 | 0.88 | 1.19 | — | 0.1 M KOH | Ag/AgCl | [33] |
| 39 | $Fe/Fe_3C$@NGL-NCNT | -0.09 | -0.7 | 1.08 | 1.78 | — | 0.1 M KOH | Ag/AgCl | [34] |
| 40 | Fe/C/N | 0.90 | 0.82 | 1.59 | 0.77 | 0.2 | 0.1 M KOH | RHE | [35] |
| 41 | $LaNiO_3$/ Nitrogen doped carbon | 0.75 | 0.64 | 1.61 | 0.97 | — | 0.1 M KOH | RHE | [36] |
| 42 | $LaNiO_3$/NCNT | 0.94 | 0.79 (900 rpm) | 1.73 (900 rpm) | 0.94 | 1.22 | 0.1 M KOH | RHE | [37] |
| 43 | NCNFs(nanoporous carbon nanofiber films) | 0.90 | 0.80 | 1.86 | 1.06 | 0.10 | 0.1 M KOH | RHE | [38] |
| 44 | Nitrogen-doped graphene/ carbon nanotube | 0.68 | 0.58 | 1.64 | 1.06 | 0.25 | 0.1 M KOH | RHE | [39] |
| 45 | 3D mesoporous graphene | 0.82 | 0.76 | 1.57 | 0.81 | — | — | RHE | [40] |
| 46 | N, P-doped carbon foam | 0.92 | 0.82 | — | — | 0.15 | 0.1 M KOH | RHE | [41] |

| | | | | | | | | | |
|---|---|---|---|---|---|---|---|---|---|
| 47 | Carbon nitrogen nanotube | 0.86 | 0.68 | 1.68 | 1.00 | — | 0.1 M KOH | RHE | [42] |
| 48 | N and P dual-doped graphene/carbon nanosheets | 0.99 | 0.91 | 1.57 | 0.66 | 0.141 | 0.1 M KOH | RHE | [43] |
| 49 | N doped carbon nanotube | 0.92 | 0.85 | 1.60 | 0.75 | 0.2 | 0.1 M KOH | RHE | [44] |
| 50 | Activated carbon sheets | -0.15 | -0.25 | 0.71 | 0.96 | — | 0.1 M KOH | SCE | [45] |

$E_{onset}$: Onset potential [V], the potential where current density reaches -0.5 mA cm$^{-2}$

$E_{-3}$: Potential at -3 mA cm$^{-2}$ or half wave potential [V]

$E_{10}$: Potential at 10 mA cm$^{-2}$ [V]

**References**

[1] A. Zhao, J. Masa, W. Xia, A. Malijusch, M-G. Willingers, G. Clavel, K. Xie, R. Schlögl, W. Schuhmann, M. Muhler, *J. Am. Chem. Soc.* 2014, **136**, 7551–7554;

[2] C. Li, X. Han, F. Cheng, Y. Hu, C. Chen, J. Chen, *Nat. Commun.* 2015, **6**, 7345;

[3] X. Cao, W. Yan, C. Jin, J. Tian, K. Ke, R. Yang, *Electrochim. Acta* 2015, **180**, 788–794;

[4] Y. J. Sa, K. Kwon, J. Y. Cheon, F. Kleitz, S. H. Joo, *J. Mater. Chem. A* 2013, **1**, 9992–10001;

[5] Y. Liu, D. C. Higgins, J. Wu, M. Fowler, Z. Chen, *Electrochem. Commun.* 2013, **34**, 125–129;

[6] S. Liu, L. Li, H. S. Ahn, A. Manthiram, *J. Mater. Chem. A* 2015, **3**, 11615–11623;

[7] X. Zhang, R. Liu, Y. Zang, G. Liu, G. Wang, Y. Zhang, H. Zhang, H. Zhao, *Chem. Commun.* 2016, **52**, 5946–5949;

[8] A. Aijaz, J. Masa, C. Rösler, W. Xia, P. Weide, A. J. R. Botz, R. A. Fischer, W. Schuhmann, M. Muhler, *Angew. Chemie Int. Ed.* 2016, **55**, 4087–4091;

[9] Y. Wang, W. Ding, S. Chen, Y. Nie, K. Xiong, Z. Wei, *Chem. Commun.* 2014, **50**, 15529–15532;

[10] M. Prabu, P. Ramakrishnan, S. Shanmugam, *Electrochem. Commun.* 2014, **41**, 59–63;

[11] P. W. Menezes, A. Indra, N. R. Sahraie, A. Bergmann, P. Strasser, M. Driess, *ChemSusChem* 2015, **8**, 164–171;

[12] X. Liu, M. Park, M. G. Kim, S. Gupta, X. Wang, G. Wu, J. Cho, *Nano Energy* 2016, **20**, 315–325;

[13] X. Ge, Y. Liu, F. W. Thomas Goh, T. S. Andy Hor, Y. Zong, P. Xiao, Z. Zhang, S. H. Lim, B. Li, X. Wang, Z. Liu, *ACS Appl. Mater. Interfaces* 2014, **6**, 12684–12691;

[14] D. Wang, X. Chen, D. G. Evans, W. Yang, *Nanoscale* 2013, **5**, 5312–5315;

[15] J. Masa, W. Xia, L. Sinev, A. Zhao, Z. Sun, S. Grützke, P. Weide, M. Muhler, W. Schuhmann, *Angew. Chemie Int. Ed.* 2014, 53, 8508–8512;

[16] S. Guo, Y. Yang, N. Liu, S. Qiao, H. Huang, Y. Liu, Z. Kang, *Sci. Bull.* 2016, **61**, 68–77;

[17] S. Mao, Z. Wen, T. Huang, Y. Hou, J. Chen, *Energy Environ. Sci.* 2014, **7**, 609–616;

[18] Y. Gorlin, D. Nordlund, T. F. Jaramillo, *ECS Trans.* 2013, **58**, 735–750;

[19] Q. Liu, J. Jin, J. Zhang, *ACS Appl. Mater. Interfaces* 2013, **5**, 5002–5008;

[20] C. Ma, N. Xu, J. Qiao, S. Jian, J. Zhang, *Int. J. Hydrogen Energy* 2016, **41**, 1–8;

[21] Z. Zhang, X. Wang, G. Cui, A. Zhang, X. Zhou, H. Xu, L. Gu, *Nanoscale* 2014, **6**, 3540–3544;

[22] M. Prabu, K. Ketpang, S. Shanmugam, *Nanoscale* 2014, **6**, 3173–3181;

[23] C. Jin, X. Cao, L. Zhang, C. Zhang, R. Yang, *J. Power Sources* 2013, **241**, 225–230;

[24] N. Ma, Y. Jia, X. Yang, X. She, L. Zhang, Z. Peng, X. Yao, D. Yang, *J. Mater. Chem. A* 2016, **4**, 6376–6384;

[25] D. Geng, N.-N. Ding, T. S. A. Hor, S. W. Chien, Z. Liu, Y. Zong, *RSC Adv.* 2015, **5**, 7280–7284;

[26] M. Shen, C. Ruan, Y. Chen, C. Jiang, K. Ai, L. Lu, *ACS Appl. Mater. Interfaces* 2015, **7**, 1207–1218;

[27] Y. Xu, W. Bian, J. Wu, J.-H. Tian, R. Yang, *Electrochim. Acta* 2015, **151**, 276–283;

[28] W. Yan, W. Bian, C. Jin, J.-H. Tian, R.Yang, *Electrochim. Acta* 2015, **177**, 65–72;

[29] W. Bian, Z. Yang, P. Strasser, R.Yang, *J. Power Sources* 2014, **250**, 196–203;

[30] W. Yan, Z. Yang, W. Bian, R. Yang, *Carbon* 2015, **92**, 74–83;

[31] W. Yan, X. Cao, J. Tian, C. Jin, K. Ke, R. Yang, *Carbon* 2016, **99**, 195–202;

[32] X. Zhao, Y. Fu, J. Wang, Y. Xu, J.-H. Tian, R. Yang, *Electrochim. Acta* 2016, **201**, 172–178;

[33] S. Ghosh, P. Kar, N. Bhandary, S. Basu, S. Sardar, T. Maiyalagan, D. Majumdar, S. K. Bhattacharya, A. Bhaumik, P. Lemmens, S. K. Pal, *Catal. Sci. Technol.* 2016, **6**, 1417–1429;

[34] J.-S. Li, S.-L. Li, Y.-J. Tang, M. Han, Z.-H. Dai, J.-C. Bao, Y.-Q. Lan, *Chem. Commun.* 2015, **51**, 2710–2713;

[35] Y. Zhao, K. Kamiya, K. Hashimoto, S. Nakanishi, *J. Phys. Chem. C* 2015, **119**, 2583–2588;

[36] W. G. Hardin, D. A. Slanac, X. Wang, S. Dai, K. P. Johnston, K. J. Stevenson, *J. Phys. Chem. Lett.* 2013, **4**, 1254–1259;

[37] D. U. Lee, H. W. Park, M. G. Park, V. Ismayilov, Z. Chen, *ACS Appl. Mater. Interfaces* 2015, **7**, 902–910;

[38] Q. Liu, Y. Wang, L. Dai, J. Yao, *Adv. Mater*. 2016, **28**, 3000–3006;

[39] G.-L. Tian, M.-Q. Zhao, D. Yu, X.-Y. Kong, J.-Q. Huang, Q. Zhang, F. Wei, *Small* 2014, **10**, 2251–2259;

[40] K. J. Lee, Y. J. Sa, H. Y. Jeong, C. W. Bielawski, S. H. Joo, H. R. Moon, *Chem. Commun.* 2015, **51**, 6773–6776;

[41] J. Zhang, Z. Zhao, Z. Xia, L. Dai, *Nat. Nanotechnol.* 2015, **10**, 444–452;

[42] R. M. Yadav, J. Wu, R. Kochandra, L. Ma, C. S. Tiwary, L. Ge, G. Ye, R. Vajtai, J. Lou, P. M. Ajayan, *ACS Appl. Mater. Interfaces* 2015, **7**, 11991–12000;

[43] R. Li, Z. Wei, X. Gou, *ACS Catal.* 2015, **5**, 4133–4142;

[44] B. Y. Xia, Y. Yan, N. Li, H. B.Wu, X. W. Lou, X. Wang, *Nat. Energy* 2016, **1**, 1–8;

[45] H. Yuan, L. Deng, X. Cai, S. Zhou, Y. Chen, Y. Yuan, *RSC Adv.* 2015, **5**, 56121–56129.
